# Supplementary material for: Simple sequence repeats drive genome plasticity and promote adaptive evolution in penaeid shrimp
Source: Commun Biol. 2021 Feb 11;4:186. doi: 10.1038/s42003-021-01716-y (PMC7878876; doi:10.1038/s42003-021-01716-y)
Supplement: Supplementary file 2 — Supplementary Information [file 42003_2021_1716_MOESM2_ESM.pdf]

Supplementary Information for

Simple sequence repeats drive genome plasticity and promote adaptive evolution in penaeid shrimp

Jianbo Yuan[1,2,3*], Xiaojun Zhang[1,2,3*], Min Wang[4,5*], Yamin Sun[6], Chengzhang Liu[1,2,3], Shihao Li[1,2,3], Yang Yu[1,2,3], Yi Gao[1,2,3], Fei Liu[1,2,3], Xiaoxi Zhang[1], Jie Kong[7], Guangyi Fan[8], Chengsong Zhang[1,2,3], Lu Feng[4,5**], Jianhai Xiang[1,2,3**], Fuhua Li[1,2,3**]

[1]CAS Key Laboratory of Experimental Marine Biology, Institute of Oceanology, Chinese Academy of Sciences, Qingdao 266071, China.

[2]Laboratory for Marine Biology and Biotechnology, Qingdao National Laboratory for Marine Science and Technology, Qingdao 266237, China.

[3]Center for Ocean Mega-Science, Chinese Academy of Sciences, Qingdao 266071, China.

[4]The Key Laboratory of Molecular Microbiology and Technology, Ministry of Education, Tianjin 300071, P. R. China.

[5]TEDA Institute of Biological Sciences and Biotechnology, Nankai University, TEDA, Tianjin 300457, P. R. China.

[6] Tianjin Biochip Corporation, Tianjin 300457, China.

[7]Key Laboratory for Sustainable Utilization of Marine Fisheries Resources of Ministry of Agriculture, Yellow Sea Fisheries Research Institute, Chinese Academy of Fishery Sciences, Qingdao 266071, China.

[8]BGI-Qingdao, BGI-Shenzhen, Qingdao 266071, China.

Supplementary Figures

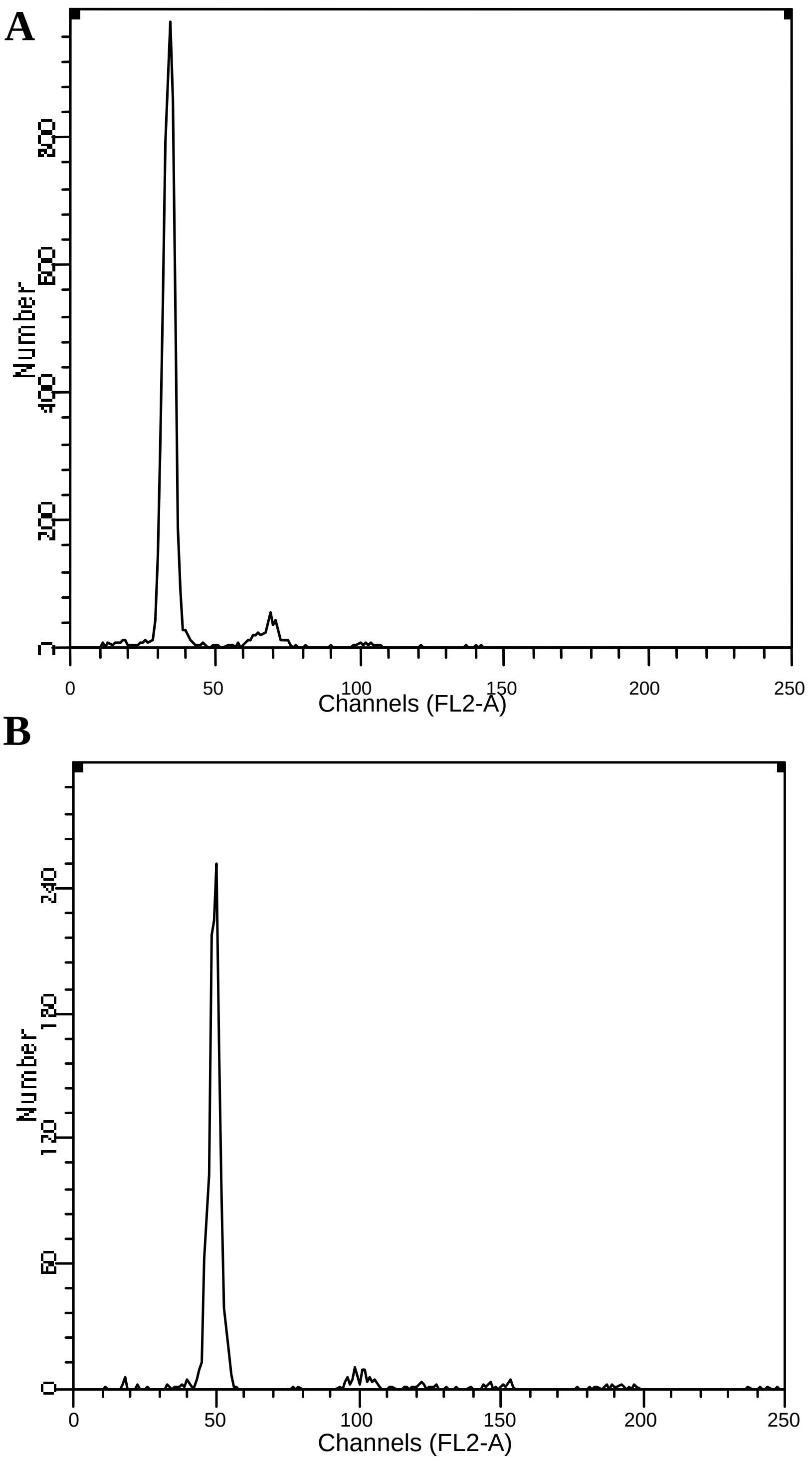

Supplementary Figure 1. Flow cytometry histogram for measuring the genome size of *F. chinensis*.

Based on the flow cytometry result, the genome size of *F. chinensis* (A) was estimated to be approximately 69.66±3.82% of the mouse genome (B) (genome size of ~2.50 Gb). Thus, the *F. chinensis* genome size identified as 1.74 Gb.

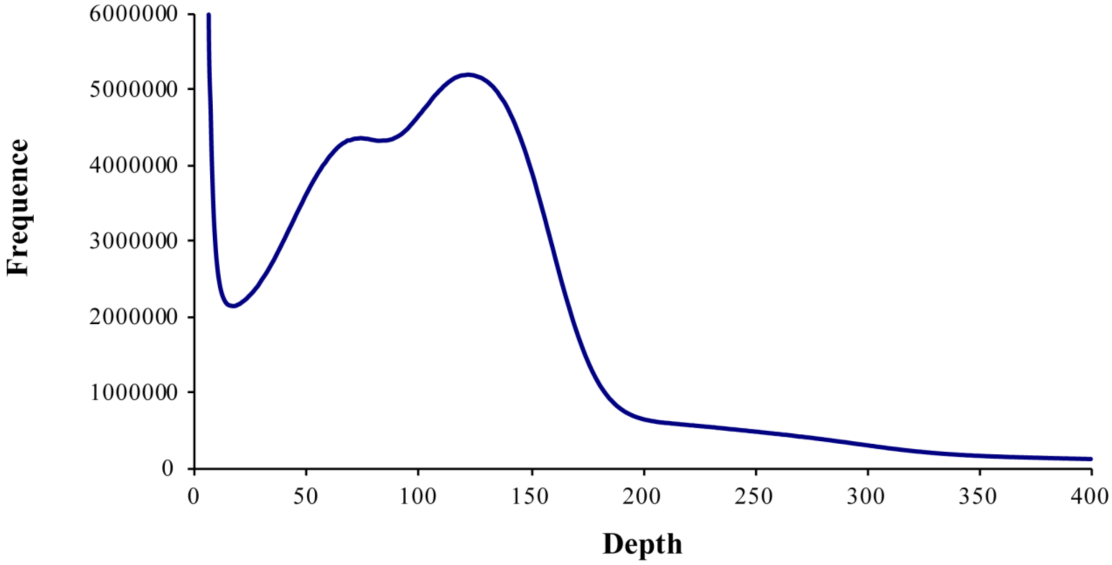

Supplementary Figure 2. K-mer distribution of the *F. chinensis* genome sequences.

K-mer analysis estimated the genome size of *F. chinensis* to be 1.88 Gb.

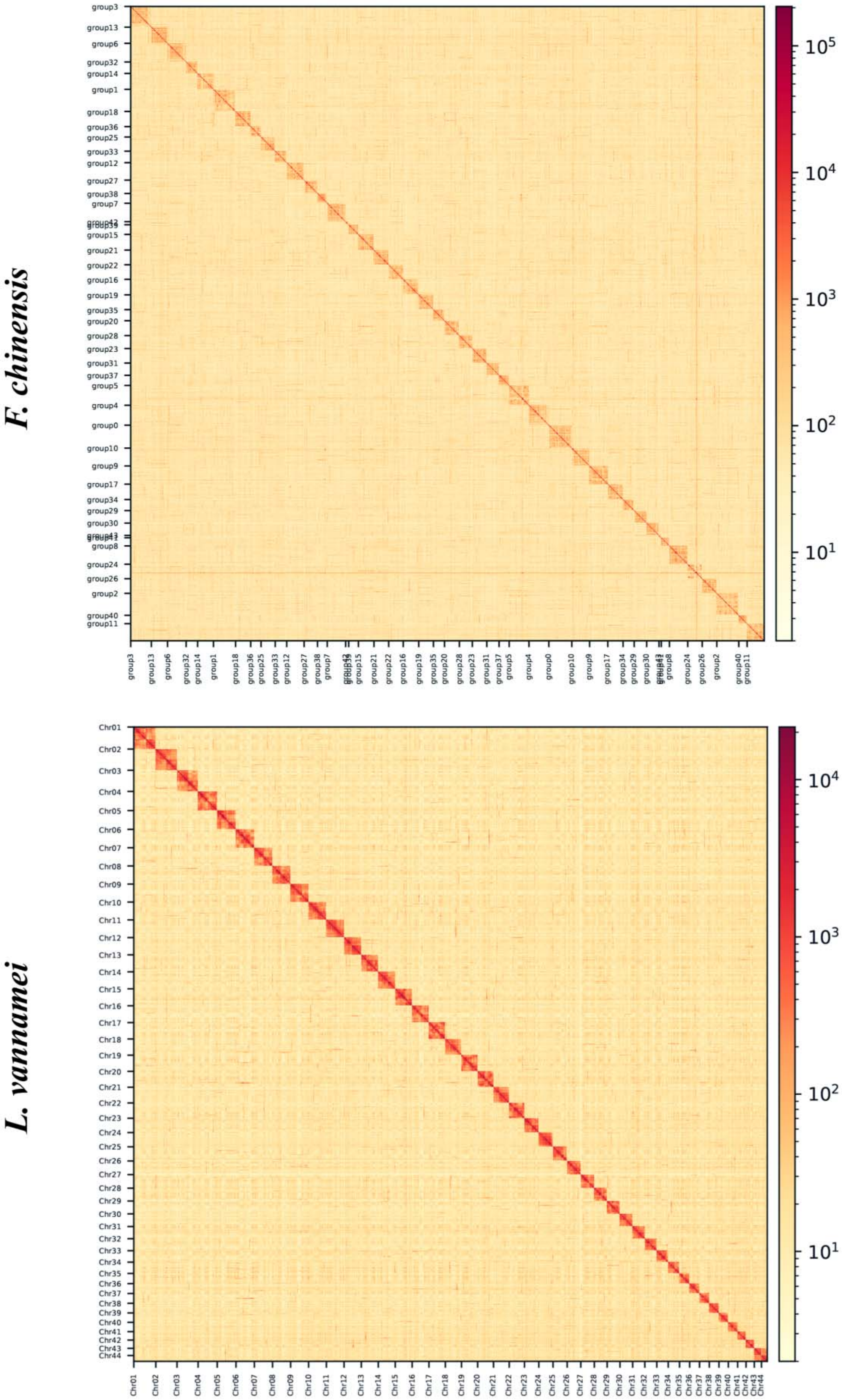

Supplementary Figure 3. Heat map of the Hi-C assembly.

The colour bar illuminates the contact density from white (low) to red (high).

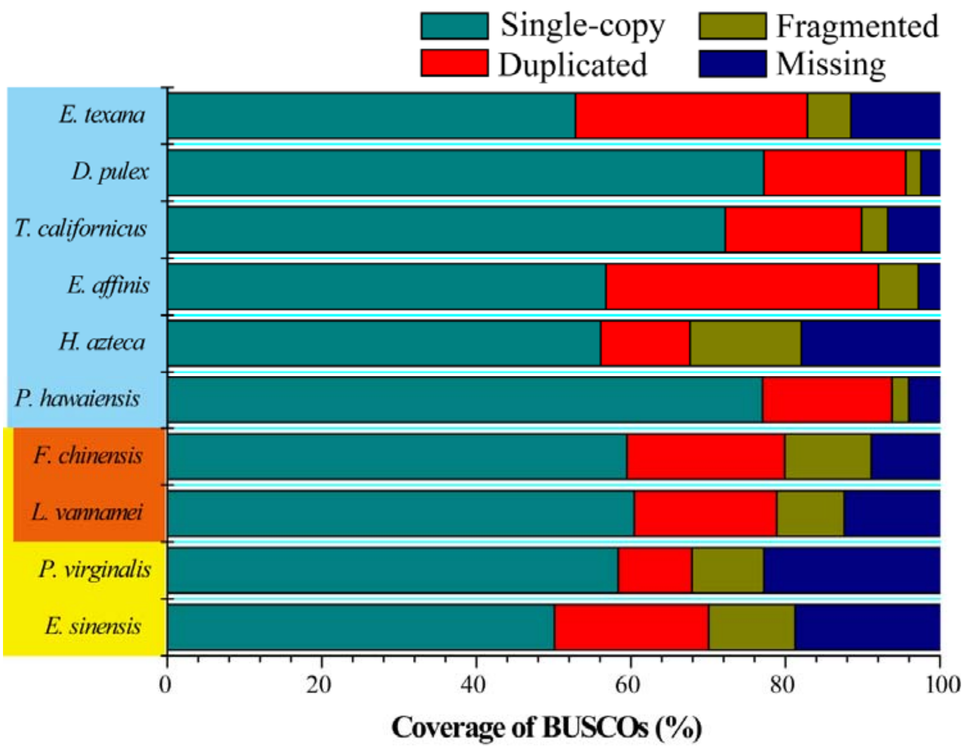

Supplementary Figure 4. The core gene coverage of crustacean genomes.

The database used for BUSCO assessment was 1066 BUSCOs of Arthropoda.

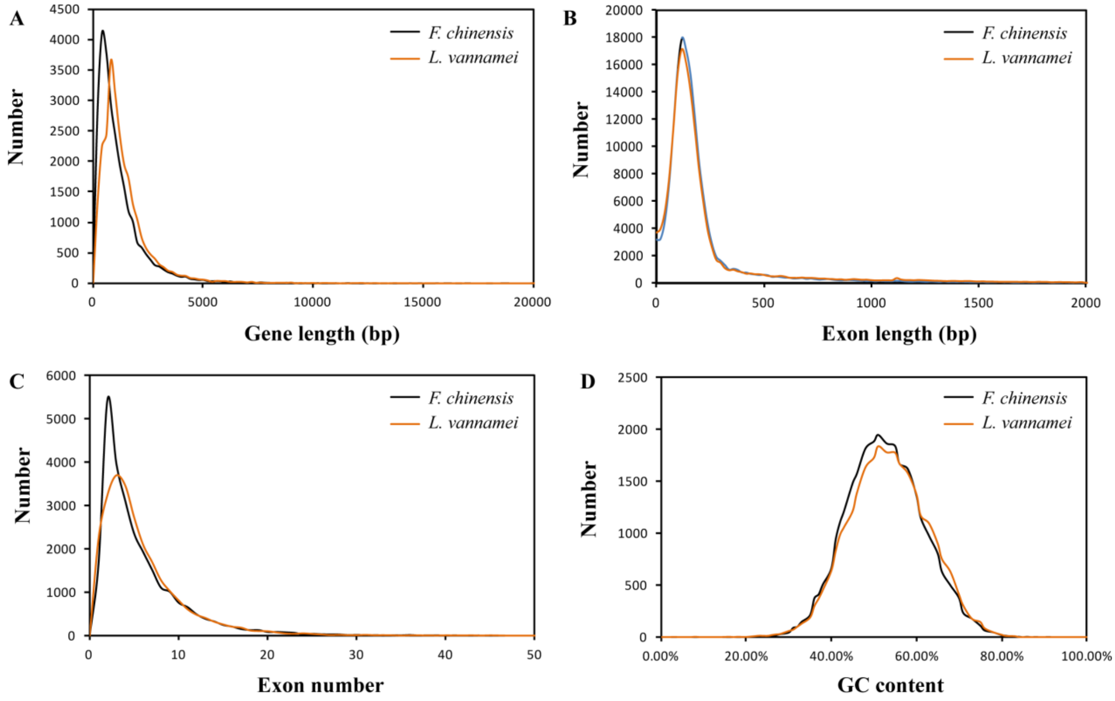

Supplementary Figure 5. Gene structure comparison between the two penaeid shrimp species.

Four gene features, including gene length, exon length, exon number, and GC content of genes, were compared between *F. chinensis* and *L. vannamei*. *F. chinensis* showed similar distribution of gene features with *L. vannamei*.

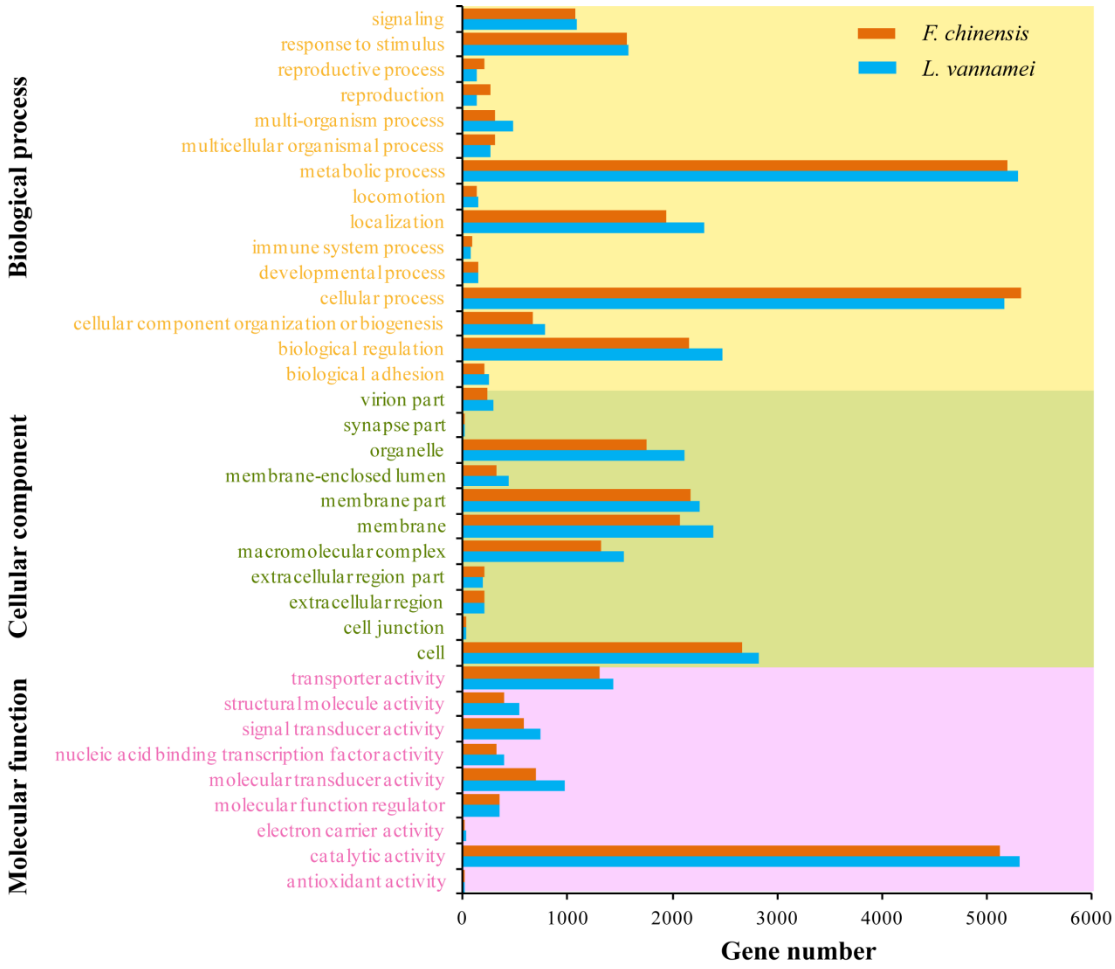

Supplementary Figure 6. Gene ontology (GO) classification of genes of the two penaeid shrimp species.

F. chinensis showed similar distribution of GO classification with *L. vannamei*. The genes of the two penaeid shrimp species were majorly distributed in GO terms of response to stimulus, metabolic process, cellular process, organelle, membrane, cell, and catalytic activity.

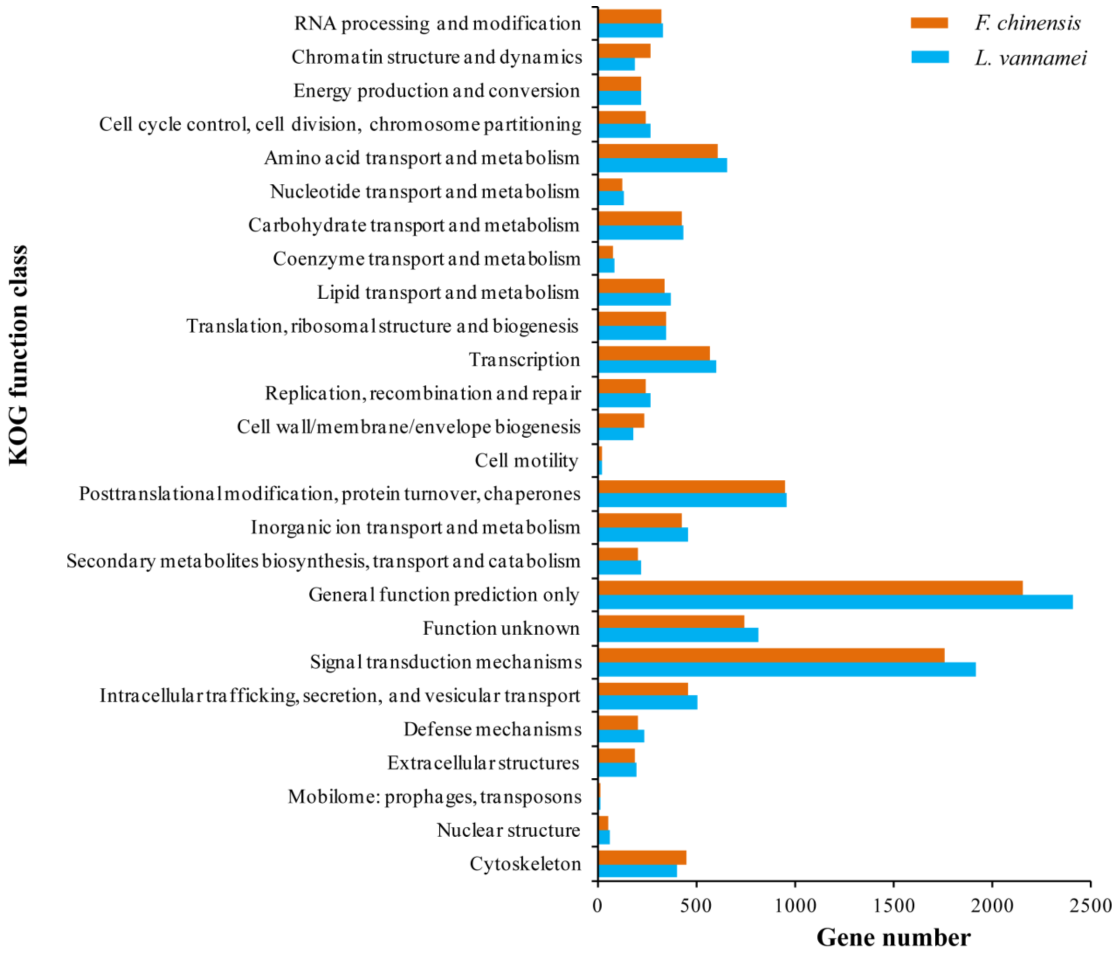

Supplementary Figure 7. KOG classification of genes of the two penaeid shrimp species.

F. chinensis showed similar distribution of KOG classification with *L. vannamei*. The genes of the two penaeid shrimp species were majorly distribute in KOG terms of cytoskeleton, signal transduction mechanisms, posttranslational modification, transcription, amino acid transport and metabolism.

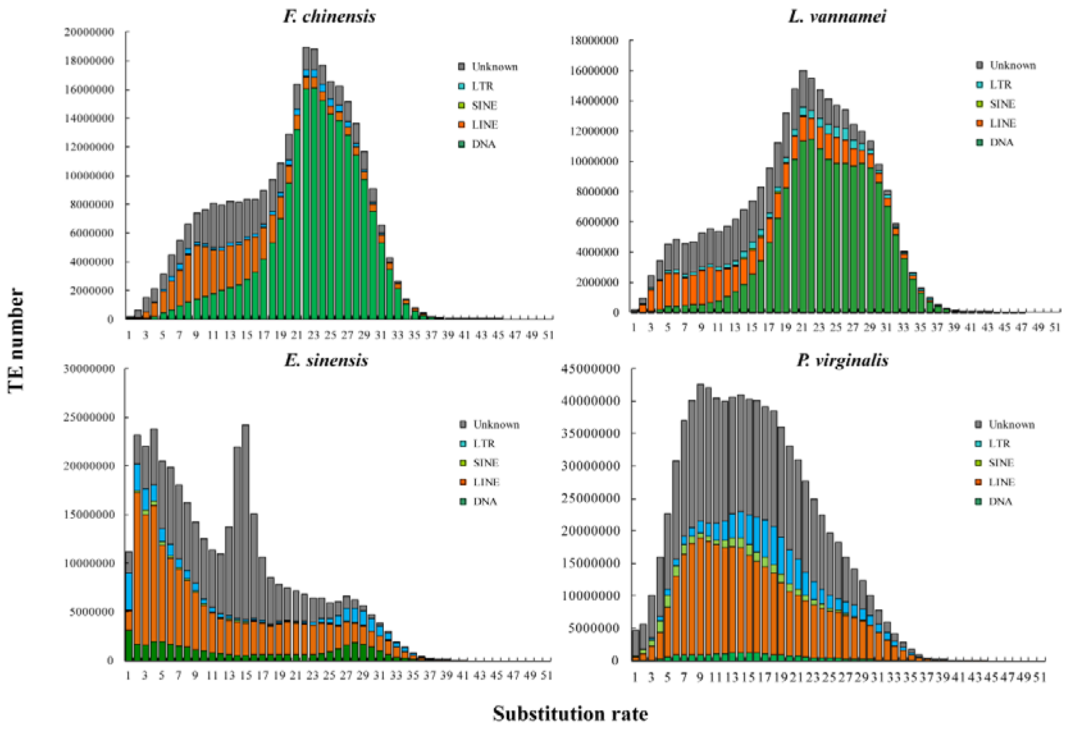

Supplementary Figure 8. Substitution rate distribution of repeats in the four decapod genomes.

The substitution rates were calculated between the genomic and repeat consensus sequences. The distribution of transposable elements of the two penaeid shrimp species was similar in comparison with the other two decapod species (*E. sinensis* and *P. virginalis*).

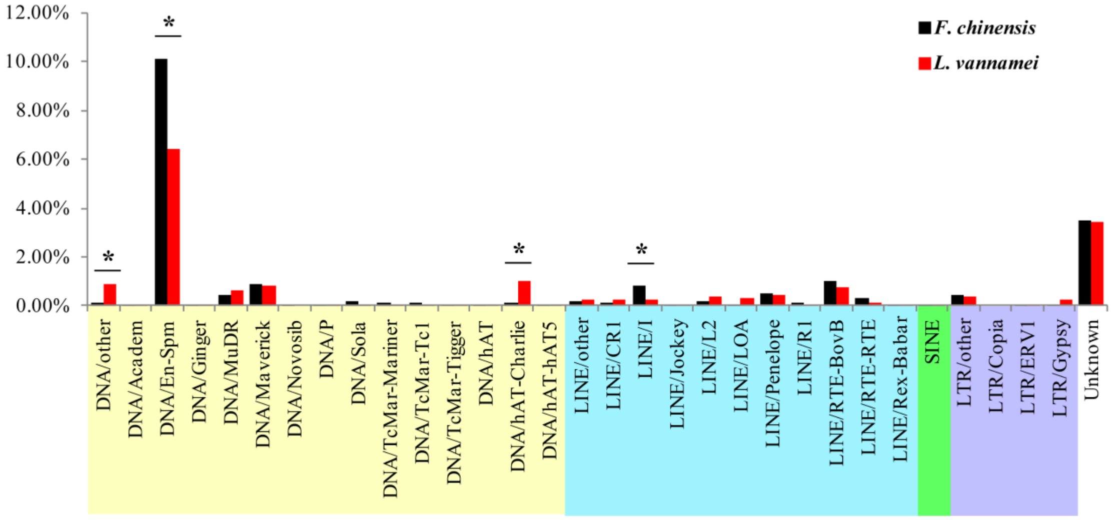

Supplementary Figure 9. Comparison of repetitive sequences between the two penaeid shrimp genomes.

The composition of transposable elements of two penaeid shrimp species was similar. However, *F. chinensis* contains more DNA transposons of En-Spm and LINE/I ($p < 0.05$). *L. vannamei* contains more DNA transposons of hAT-Charlie.

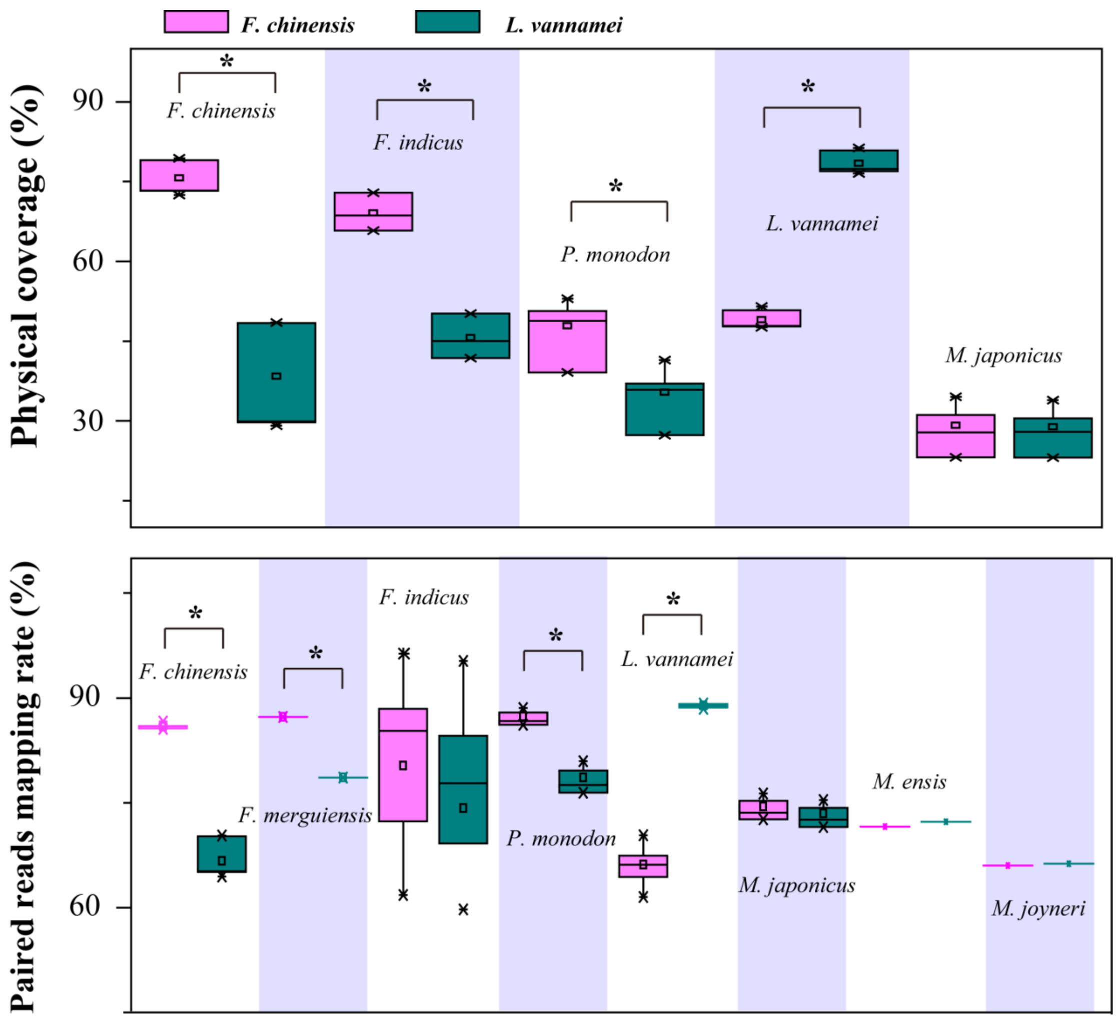

Supplementary Figure 10. Physical coverage and paired reads mapping rate of the Illumina sequencing data on the two penaeid shrimp genomes.

The genome data of the penaeid shrimp species were downloaded from NCBI SRA database (Supplementary materials Table S14). The paired-end Illumina sequencing reads were mapped against the *F. chinensis* and *L. vannamei* genomes, respectively. The physical coverage of the genome and the paired reads mapping rates were calculated. As the sequencing data of *F. indicus*, *M. ensis*, and *M. joyneri* were too scarce to cover the genome, the physical coverage of these three species did not calculated. * indicates significant difference with $p < 0.05$.

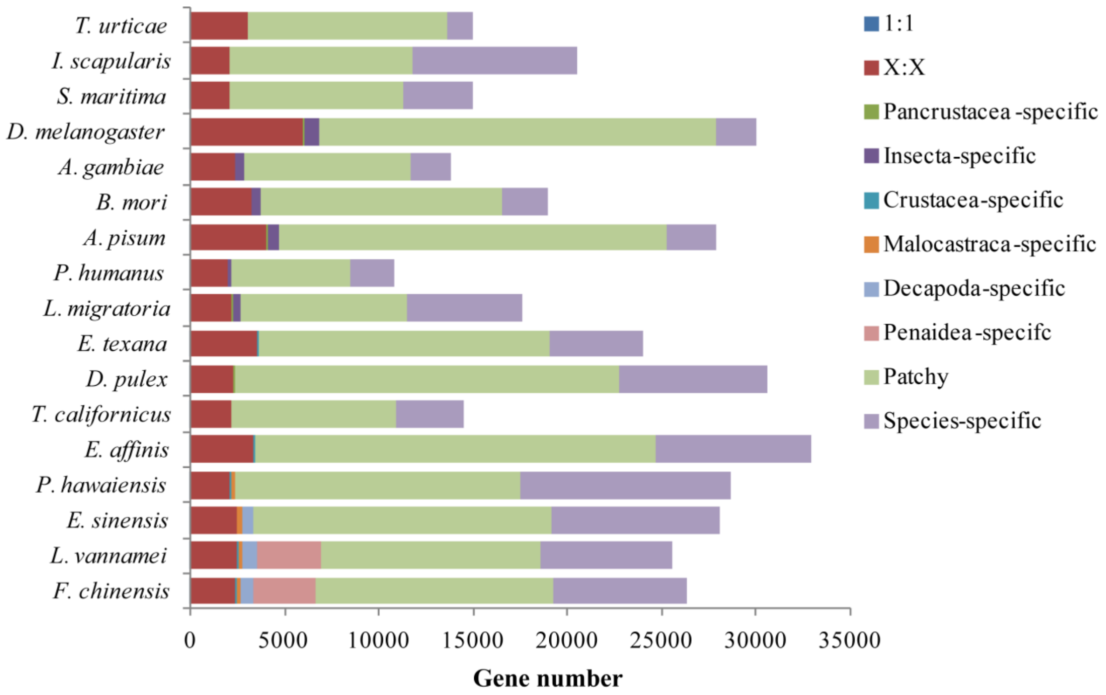

Supplementary Figure 11. Comparison of the gene repertoire of 17 arthropod genomes.

“1:1” indicates single-copy genes, “X:X” indicates orthologous genes present in multiple copies in all the ten species, where X means one or more orthologs per species, “patchy” indicates the existence of other orthologs that are presented in at least one genome.

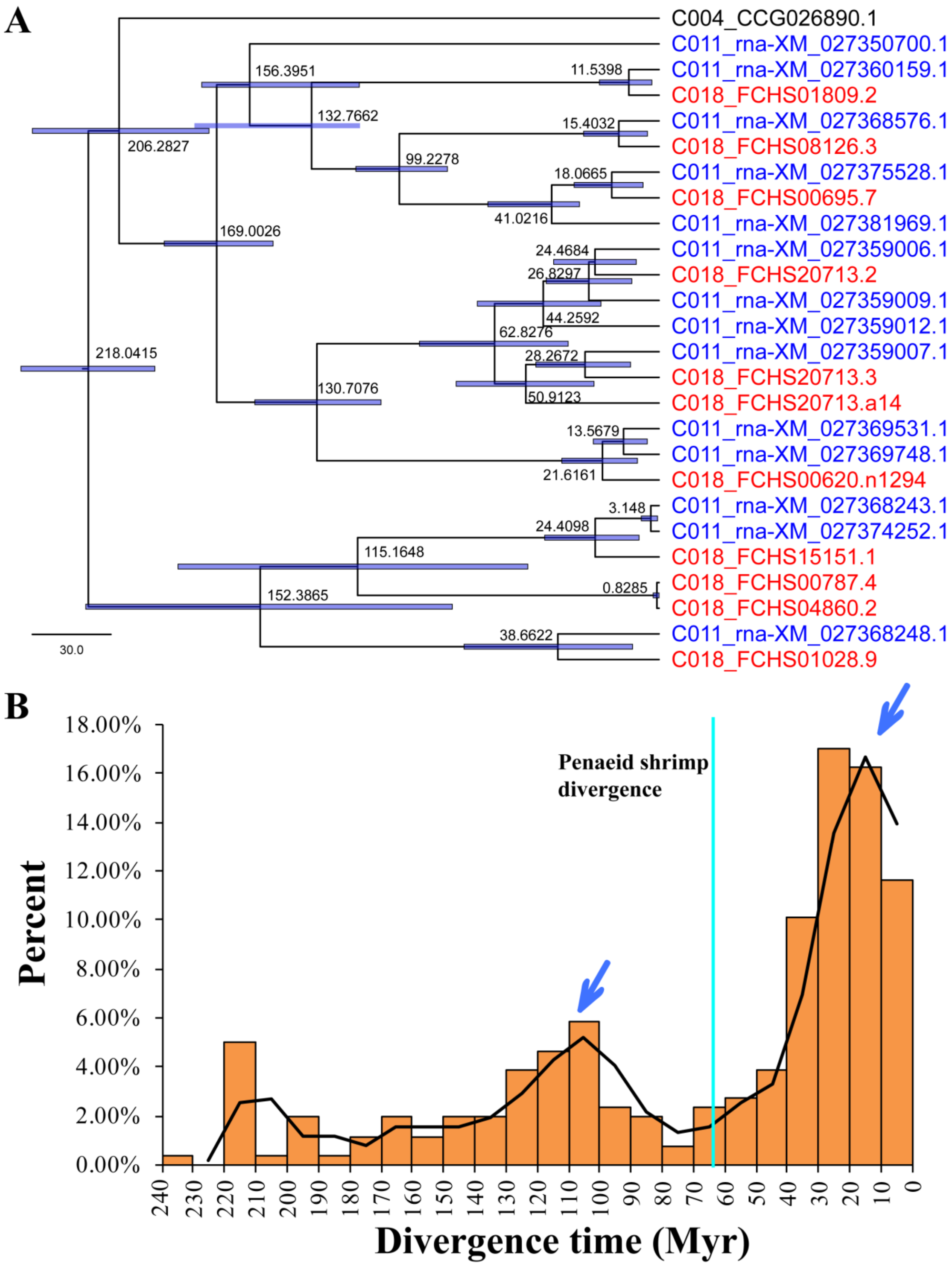

Supplementary Figure 12. The divergence times of the expanded gene families. (A) Phylogenetic tree of a expanded gene family (cytochrome P450 family 2 subfamily J) in *F. chinensis* (C018) and *L. vannamei* (C011). The numbers on the branches indicate the estimated divergence times (MYA). Error bars indicate 95% confidence levels. (B) The divergence time distribution of the expanded gene families. These genes were expanded two times (0-50 Myr and 80-160 Myr) that before and after the divergence of penaeid shrimp, respectively.

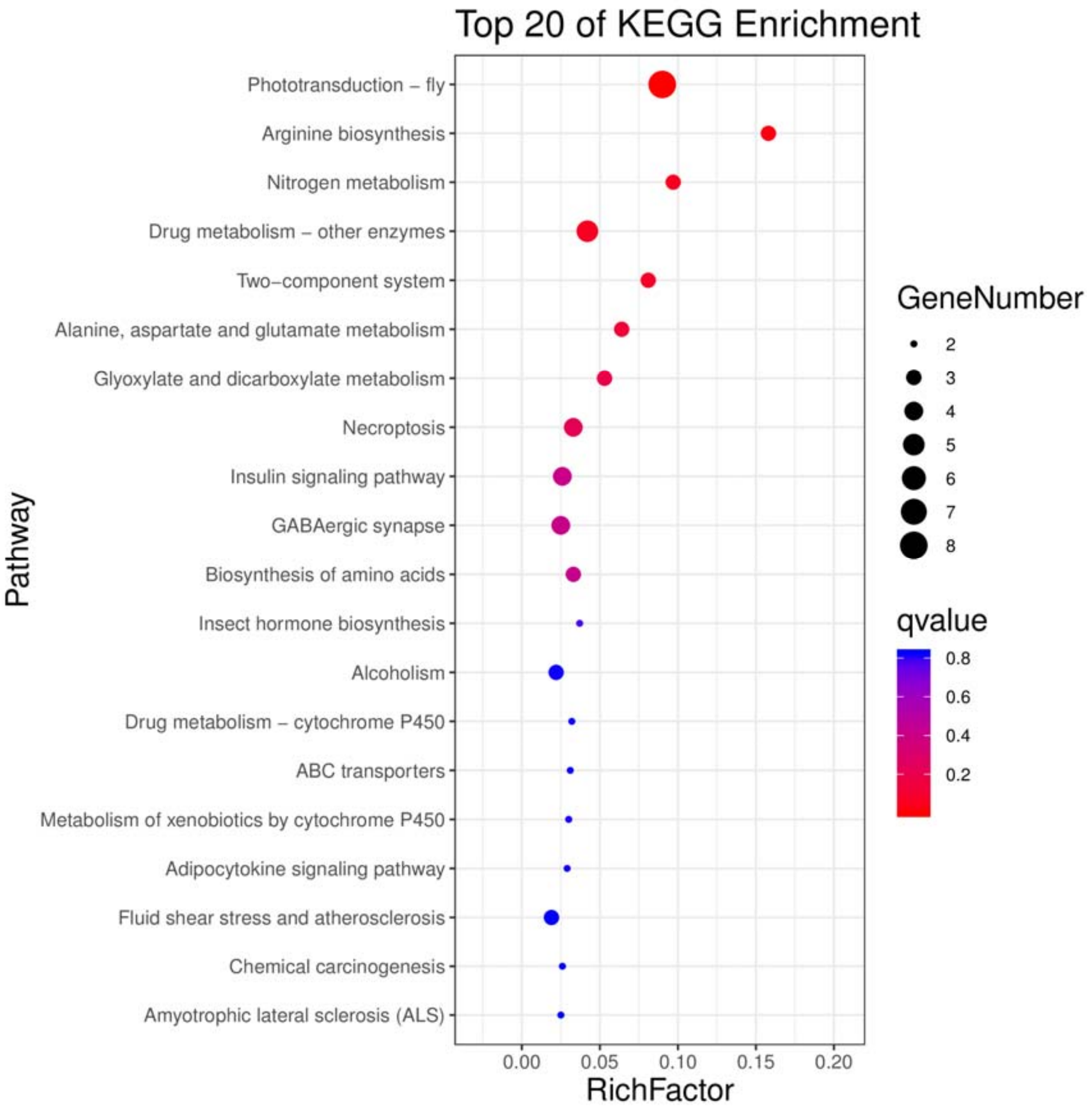

Supplementary Figure 13. KEGG enrichment analysis of the positively selected genes in penaeid shrimp.

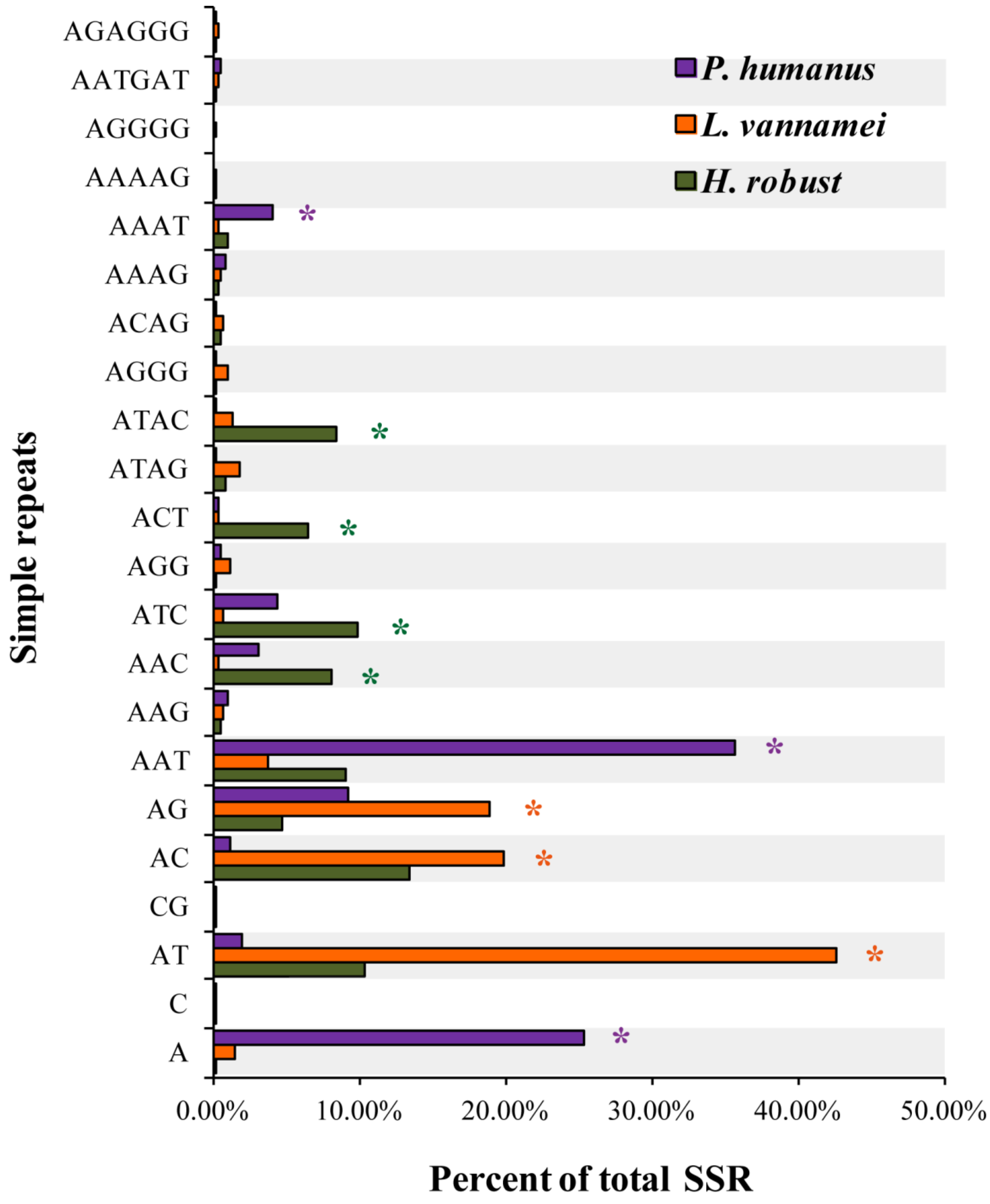

Supplementary Figure 14. Comparison of the SSR composition of SSR-rich genomes.

The penaeid shrimp harbored many more dinucleotide SSRs ($(AT)_n$, $(AC)_n$, $(AG)_n$), while *P. humanus* had more A-rich SSRs ($(A)_n$, $(AAT)_n$, $(AAAT)_n$), and *H. robusta* had more triplet and tetranucleotide SSRs ($(ATAC)_n$, $(ATC)_n$, $(ATC)_n$, and $(AAC)_n$)($p < 0.05$).

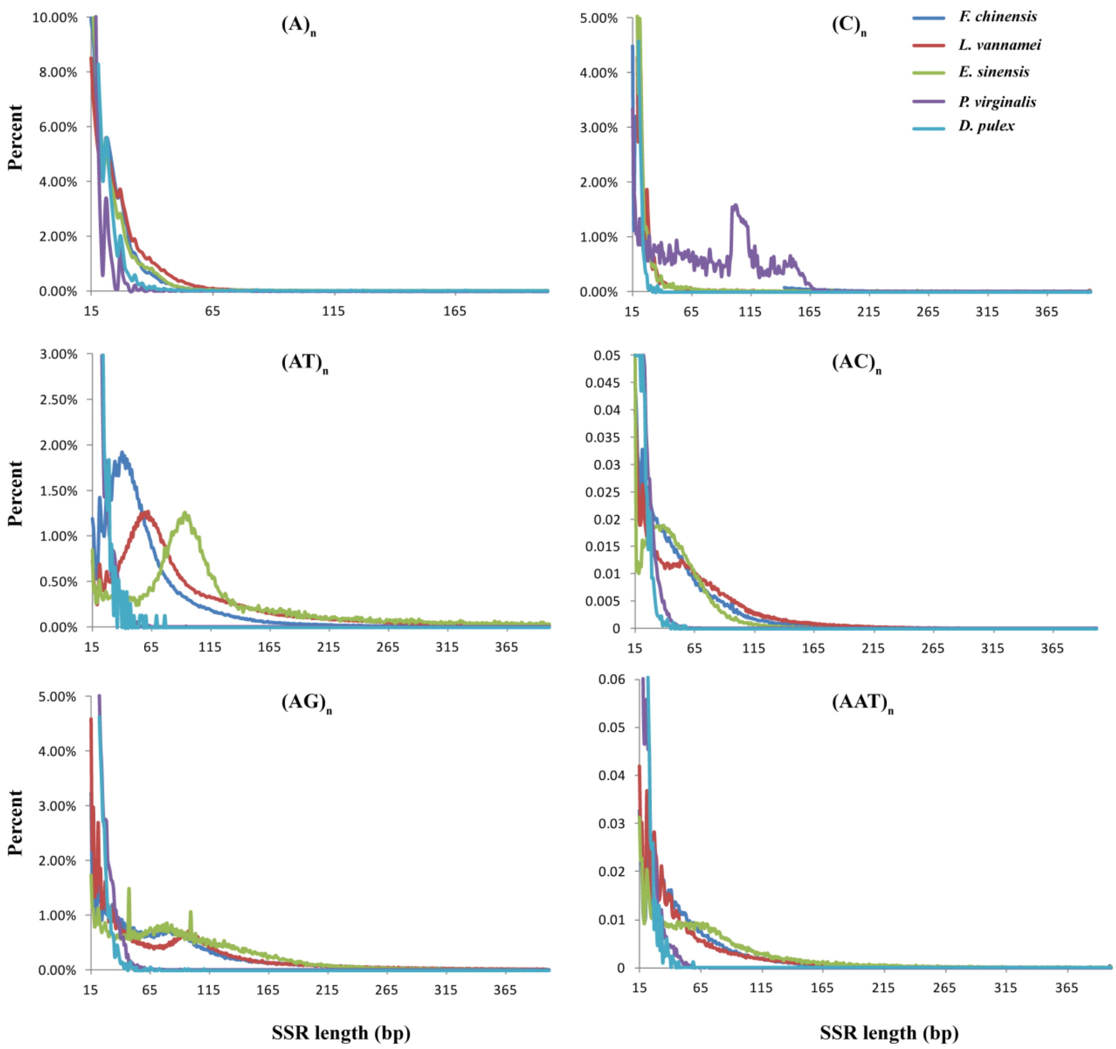

Supplementary Figure 15. SSR length distribution of the five crustacean genomes.

A peak of long SSRs could be observed along the $(AT)_n$, $(AC)_n$, and $(AG)_n$ SSRs distribution of the two penaeid shrimp species, but not present in other types of SSRs.

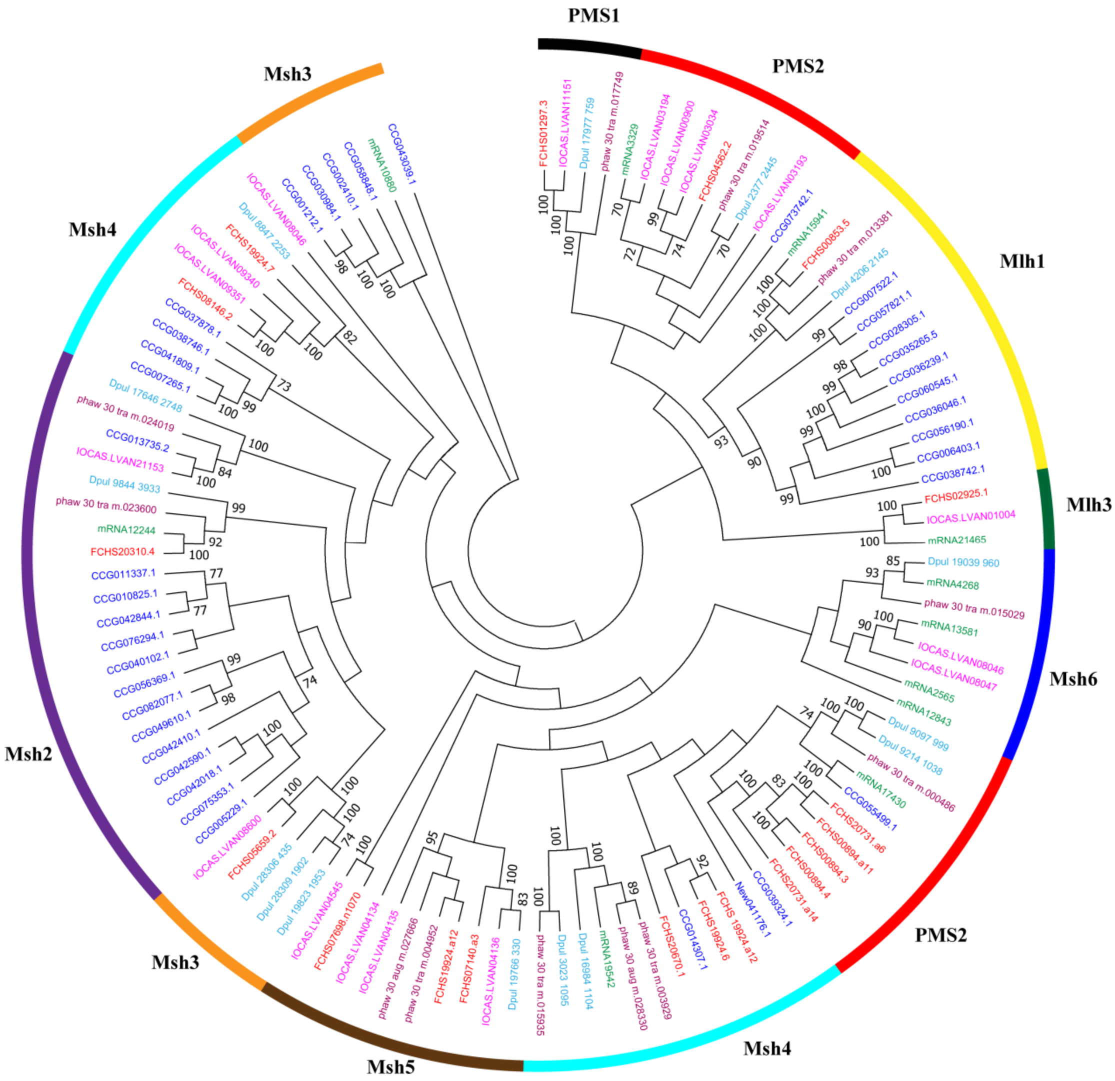

Supplementary Figure 16. Phylogenetic tree of the MMR gene family.

The phylogenetic tree was constructed by using ML methods with the substitute model of JTT+G, and 1000 bootstraps were performed. Various MMR genes were clustered together, and penaeid shrimp genomes contained most of them.

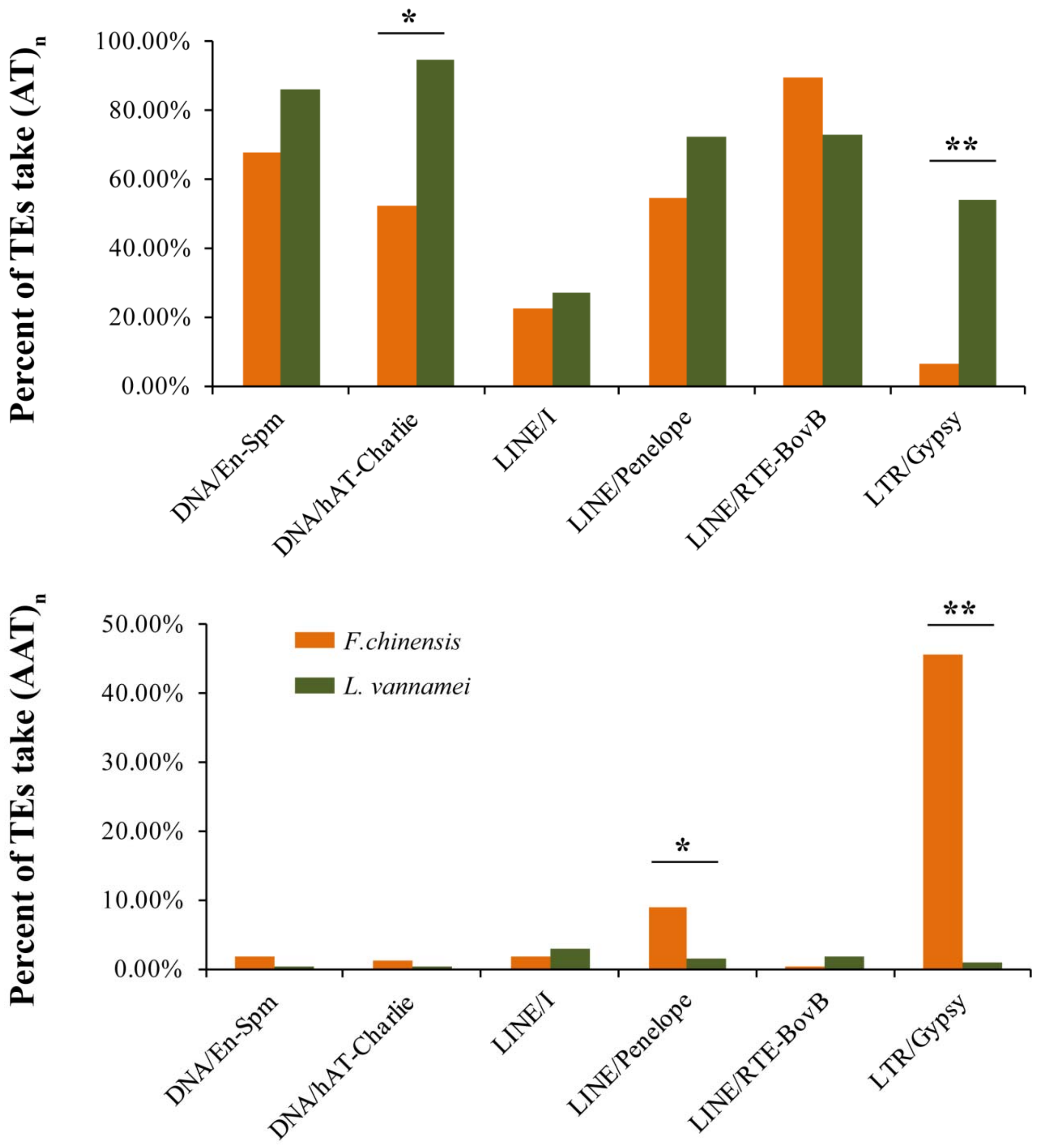

Supplementary Figure 17. Comparison of TEs harbor $(AT)_n$ and $(AAT)_n$ SSRs in the two shrimp genomes.

The percent of TEs harboring $(AT)_n$ and $(AAT)_n$ SSRs showed some differences (hAT-Charlie, Penelope and Gypsy) in two penaeid shrimp genomes. * indicates $p < 0.05$ and ** indicates $p < 0.01$. However, these TEs did not specifically harbor $(AT)_n$ and $(AAT)_n$ except Gypsy. Gypsy specifically harbor $(AT)_n$ in *L. vannamei*, while it harbor $(AAT)_n$ in *F. chinensis* specifically. Whereas hAT-Charlie and Penelope majorly contained $(AT)_n$ in both shrimp genomes.

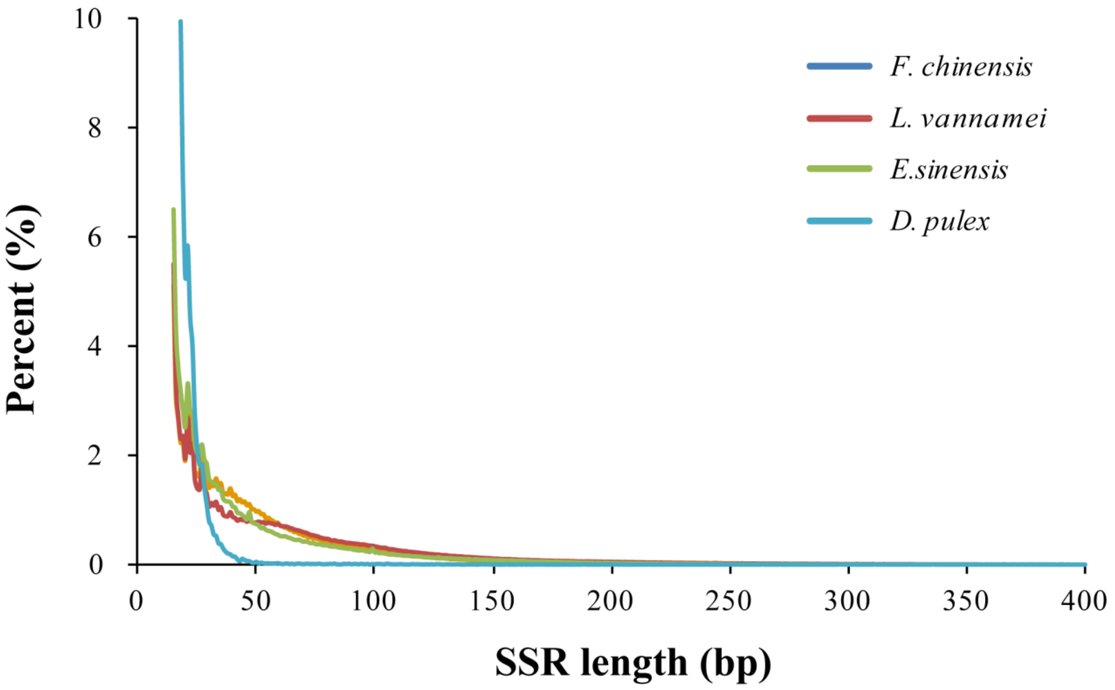

Supplementary Figure 18. SSR length distribution of four crustacean genomes. The SSRs were mostly short SSRs with the length short than 25 bp. No peak could be observed around the length of 60 bp (long SSRs).

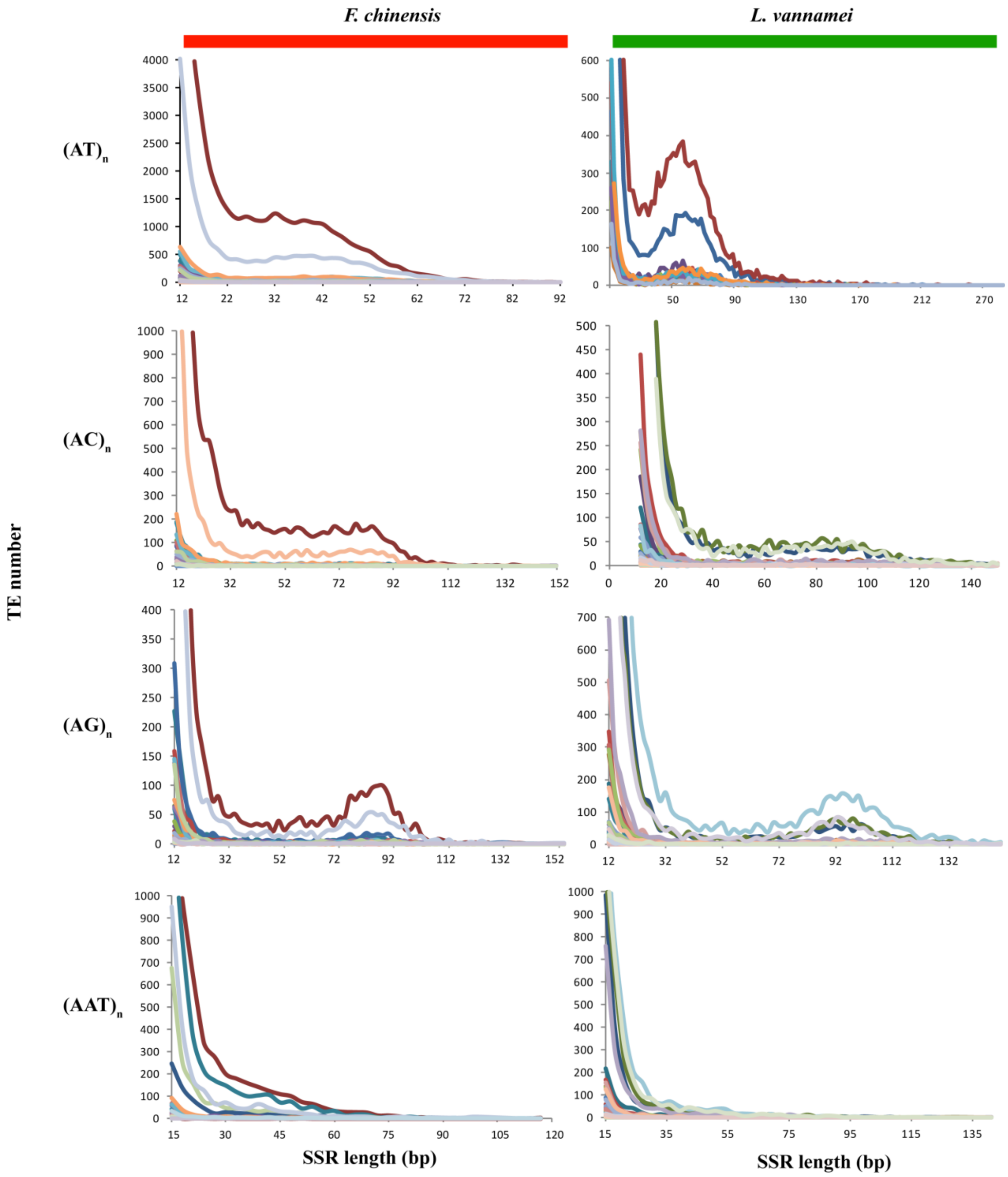

Supplementary Figure 19. The distribution of TE numbers that harboring various lengths of SSRs.

The curve with different colour indicates different TEs that harboring SSRs. Except for $(AAT)_n$ SSRs, a peak of long SSRs could be observed along the $(AT)_n$, $(AC)_n$, and $(AG)_n$ SSRs distribution in the two penaeid shrimp genomes.

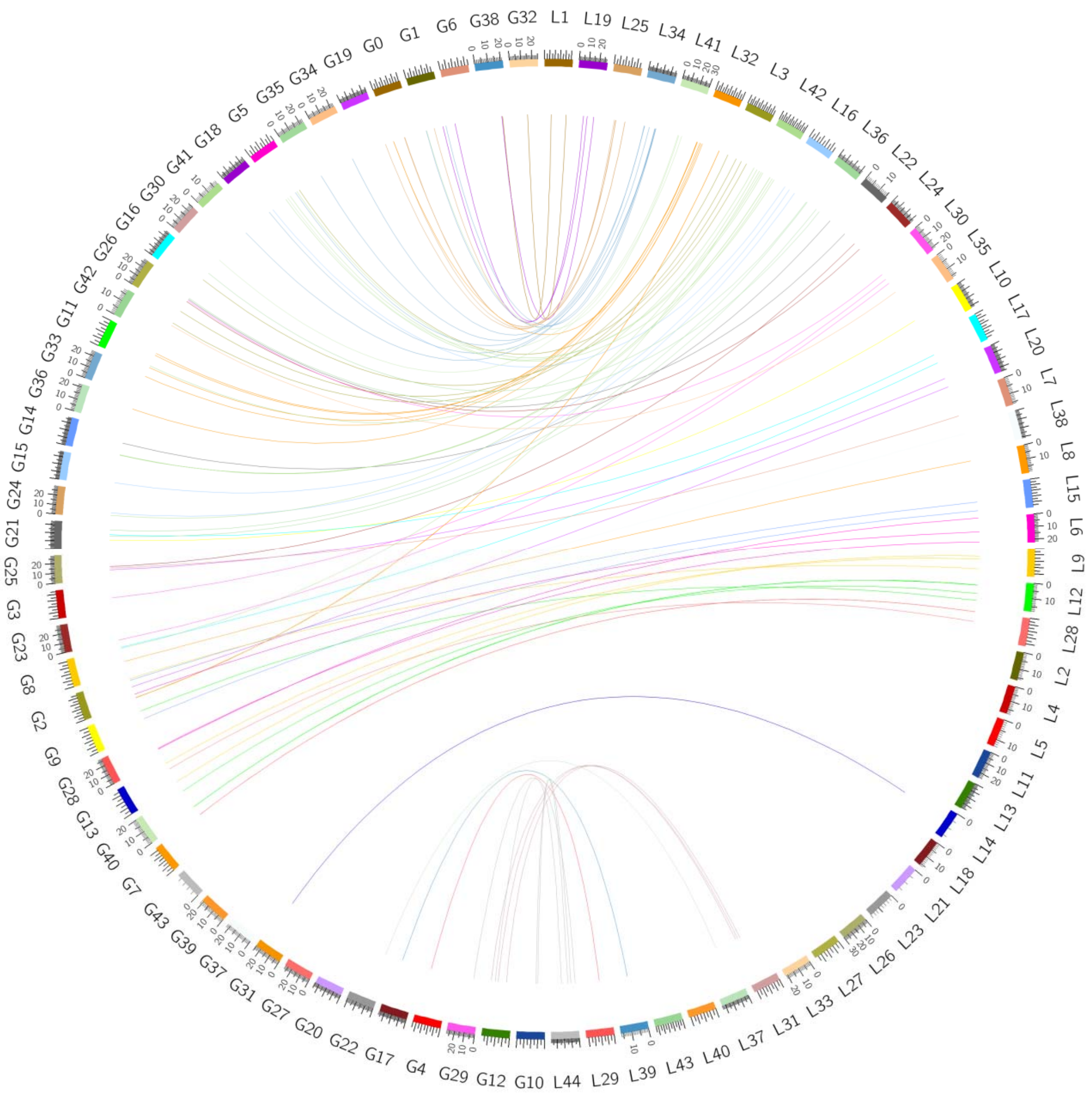

Supplementary Figure 20. The synteny of the two penaeid shrimp genomes.

The synteny of the two shrimp genomes was assessed by MCScanX. Each linking line in the center of the circle indicates a synteny block that involving at least 5 collinear gene pairs. Only 293 synteny blocks covering 2149 genes were identified between the two shrimp genomes.

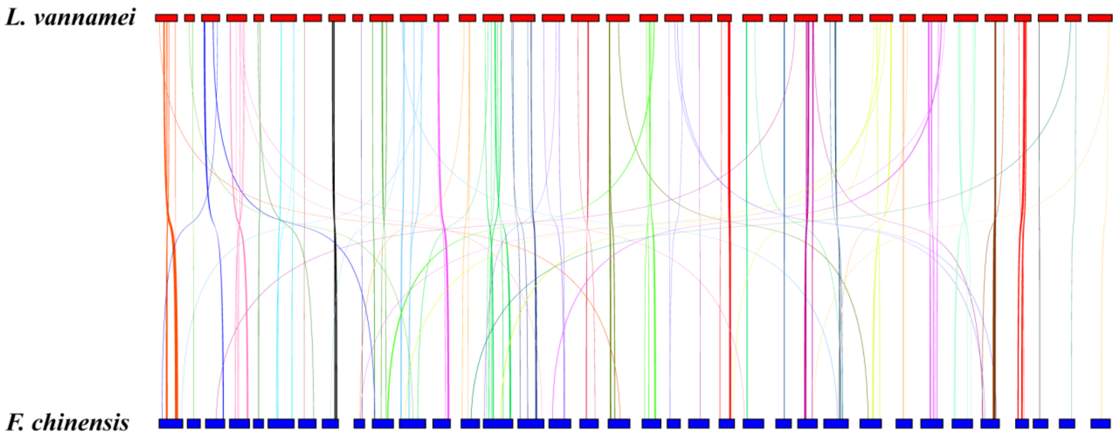

Supplementary Figure 21. The synteny of the orthologous genes of the two penaeid shrimp genomes.

Each line linked chromosomes of two shrimp genomes indicate a single orthologous gene. The chromosomes of the two shrimp species showed highly one-on-one syteny. However, the positions of orthologous genes altered their positions intrachromosome.

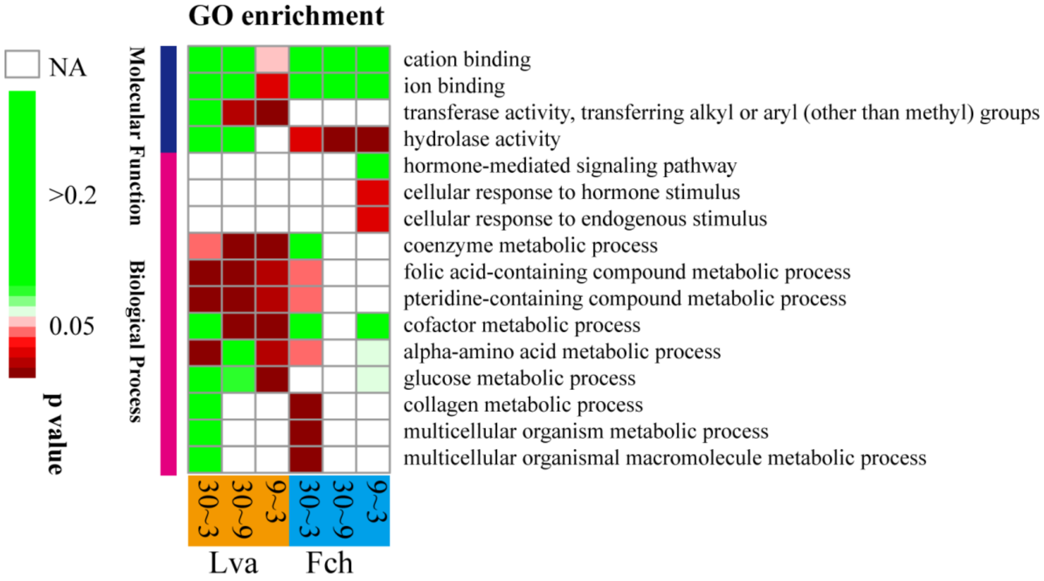

Supplementary Figure 22. GO enrichment analysis of the differential expressed genes under low-salinity pressure in the two penaeid shrimp species.

Differential gene expression analysis was performed on the two penaeid shrimp species, *L. vannmaie* (Lva) and *F. chinensis* (Fch), and the significance *p* value was calculated for the GO enrichment analysis. NA indicates no DEGs identified in relative GO terms.

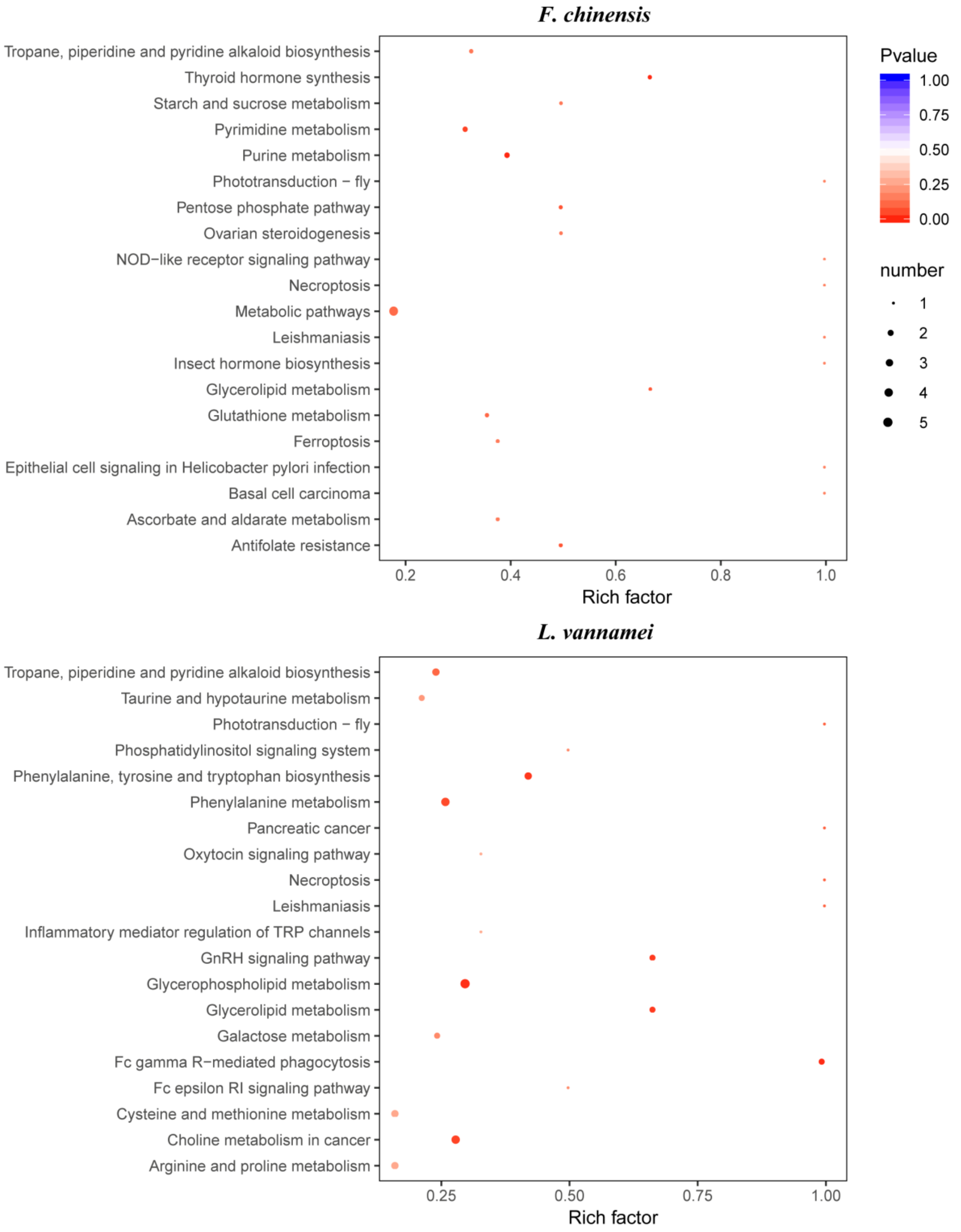

Supplementary Figure 23. KEGG enrichment analysis of the differential regulated metabolites under low-salinity pressure in penaeid shrimp.

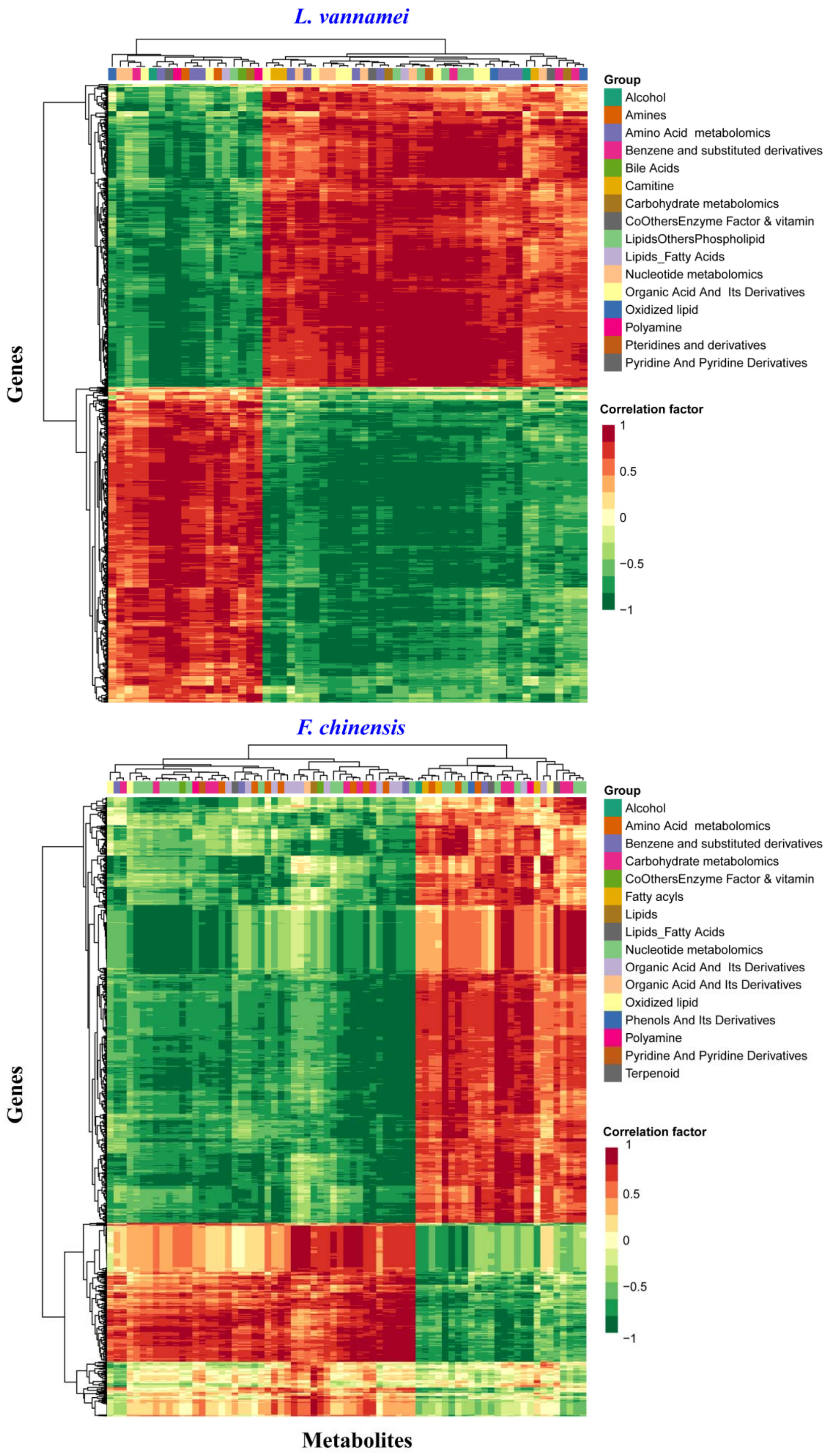

Supplementary Figure 24. The correlation of the gene expression with the metabolites under low-salinity pressure.

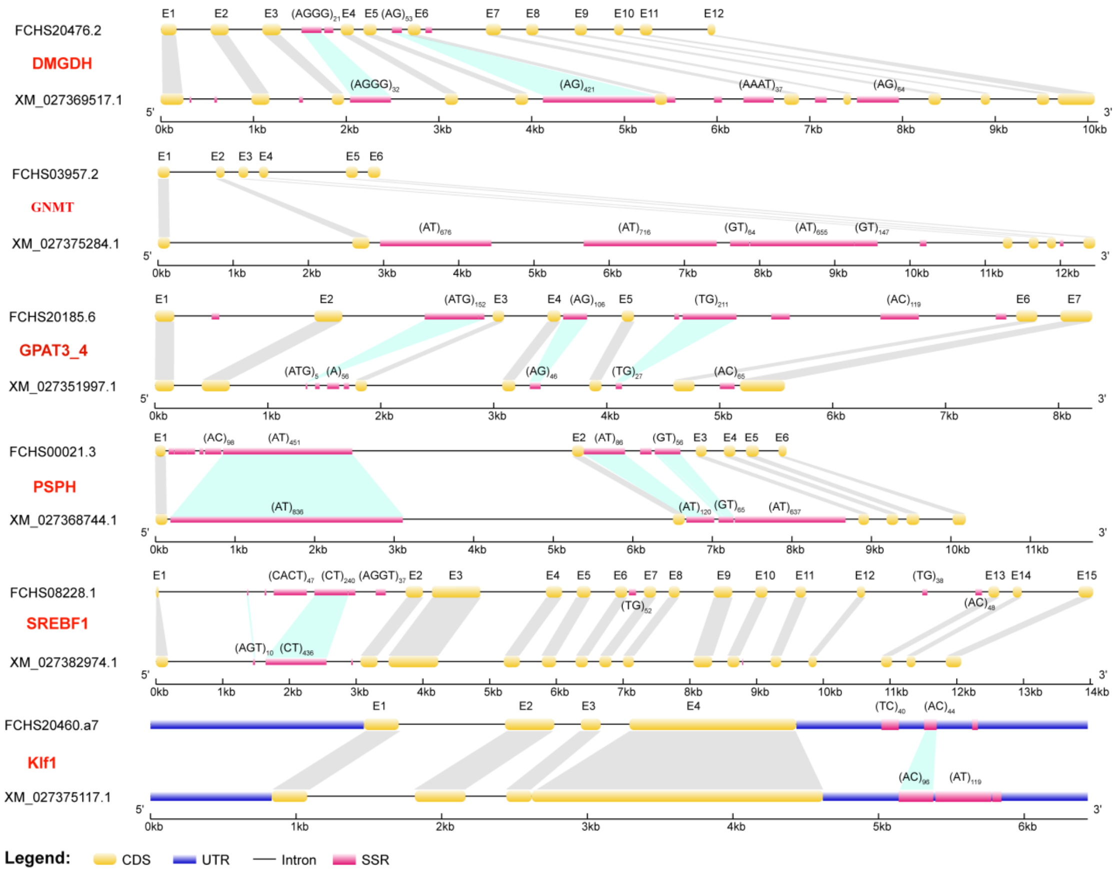

Supplementary Figure 25. The synteny of the orthologous genes in the pathway of glycine, serine and threonine metabolism between the two shrimp species.

The orthologous genes between *F. chinensis* and *L.vannamei* showed highly synteny in exons and some SSRs in introns. However, most of the SSRs in the gene body showed significant differences between the two shrimp species in all of these orthologous genes. SSR elongation and insertion could be detected when comparing the orthologous genes. Even for the genes with no SSR inserted in gene body (*Klf1*), the SSRs located in the up- or downstream showed some differences.

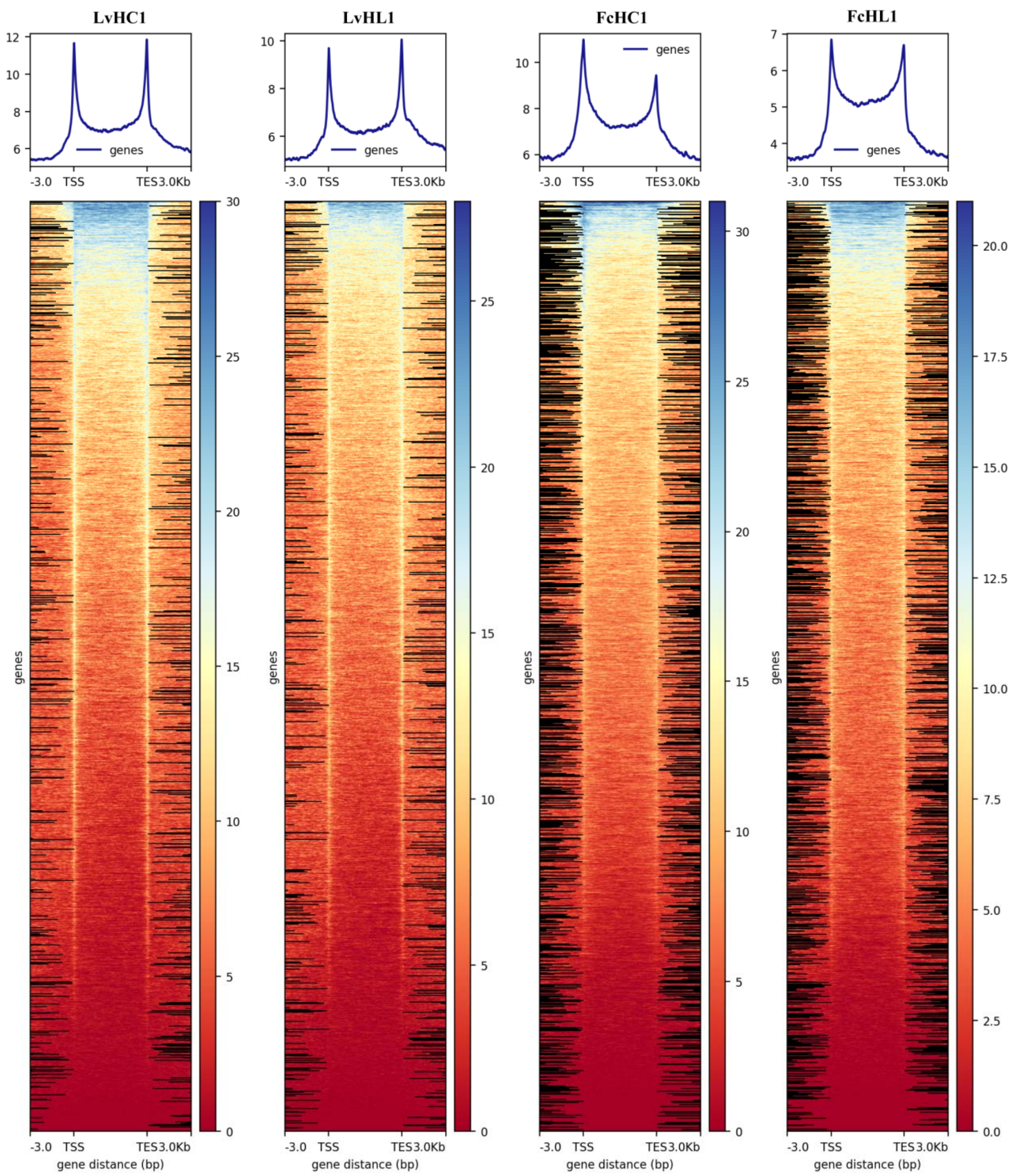

Supplementary Figure 26. The ATAC-seq mapping depth of the gene body and promoter.

The ATAC-seq reads mapping depth was calculated for each gene, and the upstream 3 Kb was considered as the promoter region. The bar of the heatmap indicates the average sequencing depth for the corresponding region. Generally, the sequencing depth was relatively higher around the transcription start site (TSS) and transcription end site (TES) than other regions.

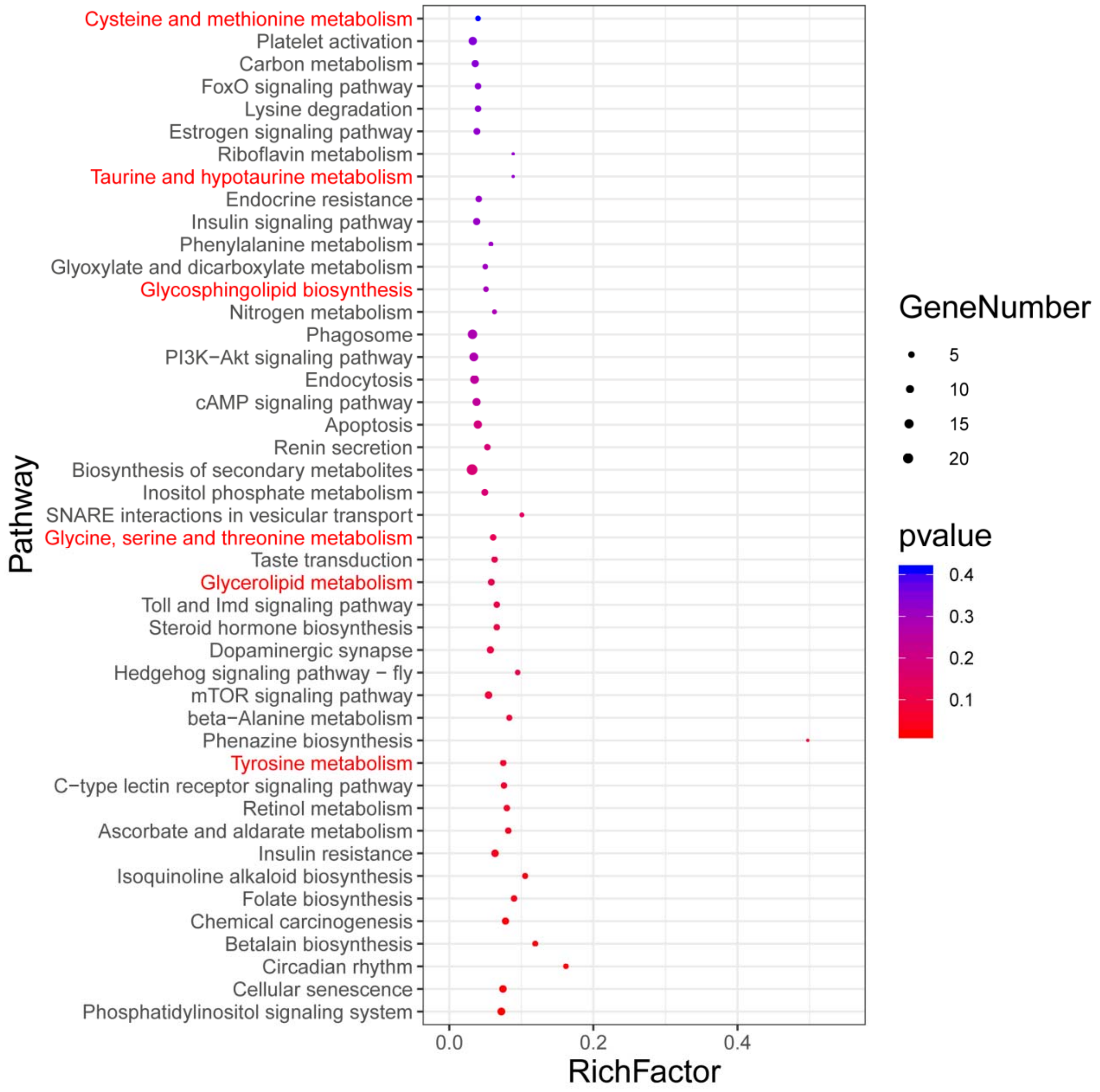

Supplementary Figure 27. The KEGG enrichment of the genes around differential ATAC peaks under low-salinity stress in ATAC-seq analysis of *L. vannamei*.

The KEGG terms in red colour were related to amino acid or lipid metabolism.

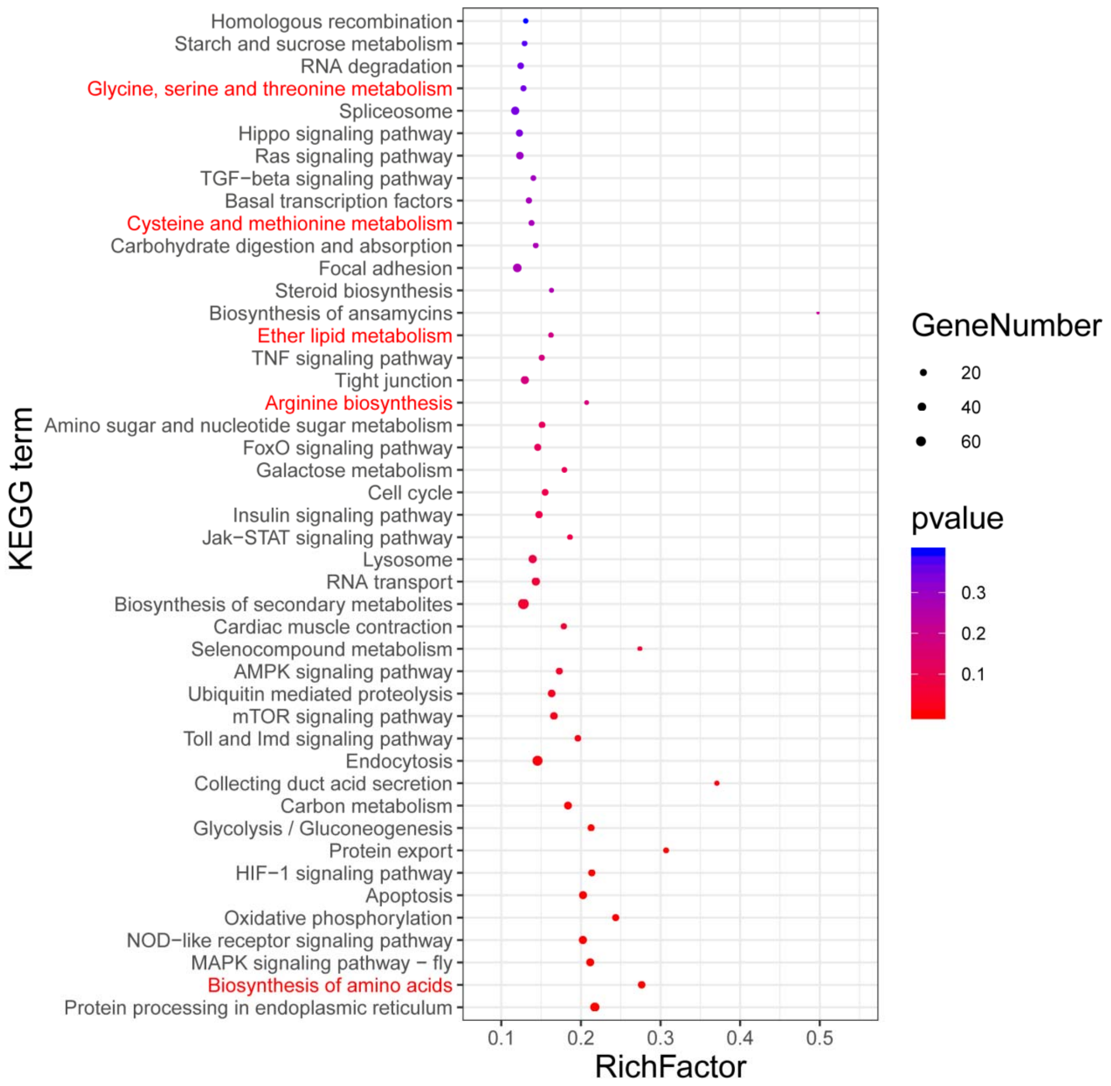

Supplementary Figure 28. The KEGG enrichment of the genes around differential peaks under low-salinity stress in ATAC-seq analysis of *F. chinensis*. The KEGG terms in red colour were related to amino acid or lipid metabolism.

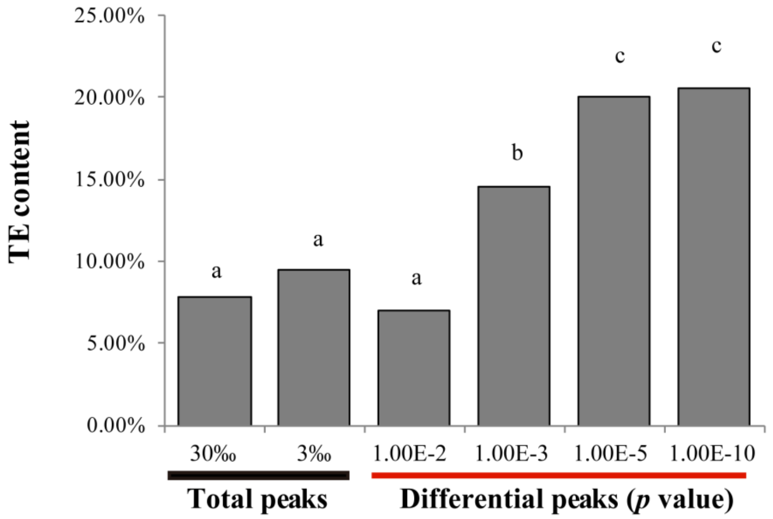

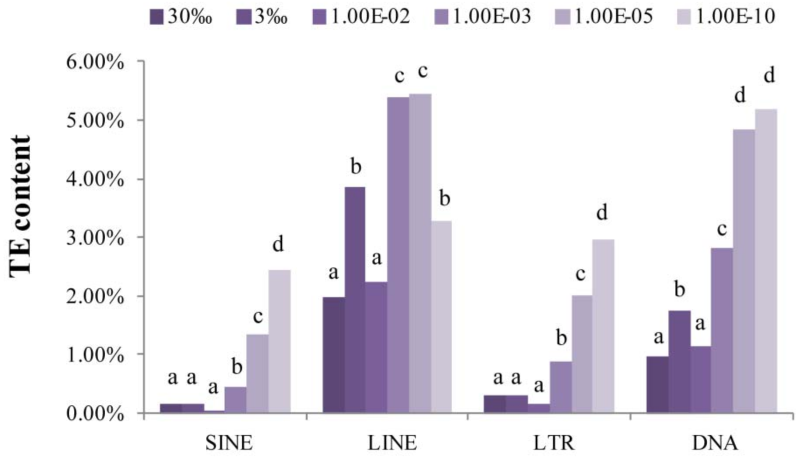

Supplementary Figure 29. TE contents in all identified peaks at 3% and 30% salinity and only the differential peaks (3% vs. 30%) identified by ATAC-seq.

The differential peaks were identified according to various *p* values from differential analyses of ATAC-seq.

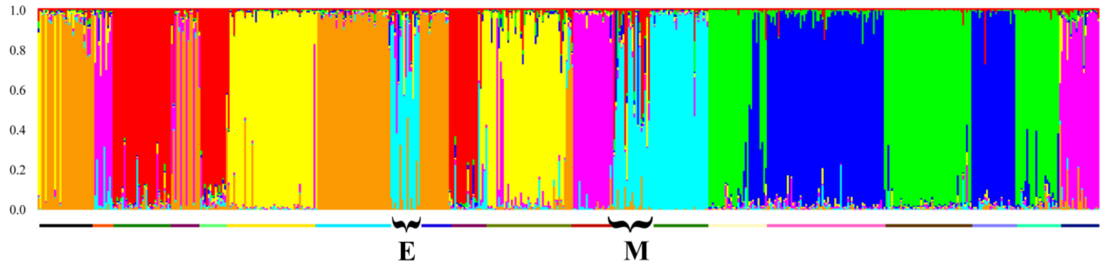

Supplementary Figure 30. The SSR polymorphism of different aquaculture populations of *L. vannamei*.

The SSR length was in highly polymorphism among different aquaculture populations (the line in various colour). E (Ecuador) and M (Mexico) were two wild-type populations.

Supplementary Tables

Supplementary Table 1. Statistics of Illumina sequencing data.

Insert Size	Reads Length	DATA	Coverage(×)*
137	100_100	94,604,750,700	50.32
170	100_100	138,076,681,910	73.45
300	100_100	37,794,515,928	20.10
500	100_100	92,431,394,740	49.17
800	100_100	38,810,731,180	20.64
2K	100_100	54,624,761,456	29.06
5K	100_100	56,626,085,318	30.12
10K	100_100	54,827,238,750	29.16
12K	100_100	32,225,310,452	17.14
17K	100_100	61,182,902,250	32.54
**Total**	**Total**	**661,204,372,684**	**351.70**

* Genome size ~1.88 Gb

Supplementary Table 2. Statistics of PacBio sequencing data.

Library	Subreads	Bases	Mean length	N50	Coverage (×)*
Fch-1	5,395,181	33,912,972,799	5,599	7,903	18.04
Fch-2	1,897,785	27,045,851,467	6,241	7,988	14.39
Fch-3	5,246,050	45,292,685,264	8,633	11,997	24.09
**Total**	**12,539,016**	**106,251,509,530**			**56.52**

* Genome size ~1.88 Gb

Supplementary Table 3. Statistics of Hi-C sequencing data.

Species	Total Base (bp)	Q20(%)	Q30(%)	Coverage (X)
F. chinensis	438,526,673,100	92.99	86.08	233.26
L. vannamei	511,576,000,000	93.48	87.16	196.76

* Genome size of *F. chinensis* is about 1.88 Gb, and Genome size of *L. vannamei* is about 2.60 Gb

Supplementary Table 4. Comparison of the two shrimp genome assemblies.

	F. chinensis		*L. vannamei*	
	**Number**	**Size**	**Number**	**Size**
Total contigs	49,957	1,554,960,535	50,304	1,618,026,442
Longest contig		829,035		739,419
Contig N50	42,892	58,996	42,878	57,650
Contig N90	21,519	13,999	20,331	14,641
Total scaffolds	8,768	1,581,129,620	28,409	1,631,536,563
Longest scaffold		45,805,217		47,298,368
Scaffold N50	22	28,916,617	21	31,296,514
Scaffold N60	28	25,750,730	27	27,178,000
Scaffold N70	34	22,083,550	33	23,191,203
Scaffold N80	77	147,111	41	17,560,698
Scaffold N90	1,744	66,757	5,575	63,975

Supplementary Table 5. Illumina reads coverage on the *F. chinensis* genome.

Reads	Number of clean reads	981,748,772
	Percentage of mapped reads	91.12%
	Percentage of Paired reads	85.70%
Genome	Coverage（%）	82.46%
	Coverage at least 5×（%）	75.34%
	Coverage at least 10×（%）	70.91%
	Coverage at least 20×（%）	64.54%
	Coverage at least 50×（%）	46.47%

Supplementary Table 6. Unigene coverage on the *F. chinensis* genome.

**Unigenes**	**Number**	**Percent**
Total unigenes	64,708	100%
Matched unigenes	61,360	94.83%
90% in one scaffold	40,156	62.06%
50% in one scaffold	54,554	84.31%

Supplementary Table 7. Assessment the genome coverage of *F. chinensis* using BUSCO.

Complete BUSCOs	891 (83.58%)
Complete and single-copy BUSCOs	785 (73.63%)
Complete and duplicated BUSCOs	106 (9.94%)
Fragmented BUSCOs	97 (9.10%)
Missing BUSCOs	78 (7.31%)
Total BUSCO groups searched	1066(100%)

Supplementary Table 8. Macrosynteny between the genome assembly by Hi-C data and linkage maps of *L. vannamei*.

LG	HIC	Length	MakerNum
LG34	1	43094143	147
LG23	2	17230118	33
LG19	5	34381183	133
LG11	6	28891236	87
LG2	7	17803676	51
LG14	8	44804749	17
LG15	8	44804749	152
LG17	9	36257752	133
LG43	10	36244676	125
LG39	11	21561385	46
LG8	12	17560698	46
LG31	13	37747505	130
LG25	15	42416622	132
LG4	16	29400107	51
LG24	17	17562319	64
LG18	18	26453084	73
LG9	19	36891603	144
LG29	21	42707934	157
LG28	22	37147500	116
LG20	23	47298368	155
LG16	24	37504298	128
LG5	25	21631114	66
LG6	26	29425547	72
LG36	27	27178000	105
LG3	28	32672231	119
LG26	29	33855442	87
LG35	30	24502028	80
LG41	31	23191203	81

LG33	32	22018822	61
LG13	33	32300476	139
LG7	35	26185043	61
LG38	36	37020464	69
LG21	37	29118703	22
LG22	37	29118703	88
LG27	38	26456696	100
LG40	39	37974538	127
LG1	40	23323404	73
LG32	41	40728778	127
LG42	42	39728044	142
LG10	43	31540936	108
LG12	44	22423333	69
LG1	45	30003605	119
LG30	47	21067011	68
LG44	49	36011199	140
LG37	51	31296514	103

Supplementary Table 9. Comparison of gene structures between the two shrimp species.

Gene structure	***F. chinensis***	***L. vannamei***
Gene number	26,343	25,596
Gene average length (bp)	7,312	8,889
Gene Max length (bp)	269,656	329,769
Gene Min length (bp)	150	165
CDS average length (bp)	1,294	1,546
CDS Max length (bp)	24,525	35,259
CDS Min length (bp)	150	160
Exon number per gene	5.77	5.94
Exon average length (bp)	224.13	259.95
Intron average length (bp)	1260.25	1483.77

Supplementary Table 10. Summary of repetitive sequences.

**Repeats**	***F. chinensis***	***L. vannamei***	***E. sinensis***	***P. virginalis***
Genome length	1.57 Gb	1.66 Gb	1.56 Gb	3.29 Gb
Repeats	48.58%	49.39%	35.57%	26.59%
DNA	13.00%	9.33%	2.30%	0.62%
DNA/Academ	0.00%	0.01%	0.00%	0.00%
DNA/En-Spm	10.08%	6.39%	0.82%	0.01%
DNA/Ginger	0.00%	0.00%	0.01%	0.00%
DNA/MuDR	0.46%	0.64%	0.03%	0.00%
DNA/Maverick	0.87%	0.80%	0.10%	0.07%
DNA/Merlin	0.02%	0.00%	0.01%	0.00%
DNA/P	0.05%	0.02%	0.08%	0.00%
DNA/Sola	0.20%	0.06%	0.00%	0.00%
DNA/TcMar-Mariner	0.12%	0.06%	0.00%	0.00%
DNA/TcMar-Tc1	0.10%	0.03%	0.02%	0.00%
DNA/TcMar-Tigger	0.00%	0.04%	0.32%	0.34%
DNA/hAT	0.04%	0.01%	0.09%	0.00%
DNA/hAT-Charlie	0.14%	1.00%	0.09%	0.03%
DNA/hAT-hAT5	0.00%	0.02%	0.00%	0.00%
LINE	3.27%	2.82%	9.72%	9.52%
LINE/CR1	0.14%	0.25%	4.06%	5.99%
LINE/I	0.84%	0.23%	0.10%	1.10%
LINE/Jockey	0.09%	0.06%	0.05%	0.29%
LINE/L2	0.17%	0.35%	0.36%	0.71%
LINE/LOA	0.04%	0.30%	0.00%	0.00%
LINE/Penelope	0.51%	0.45%	0.04%	0.74%
LINE/R2	0.10%	0.01%	0.00%	0.00%
LINE/RTE-BovB	0.99%	0.77%	0.91%	0.12%
LINE/RTE-RTE	0.35%	0.15%	0.07%	0.05%
LINE/Rex-Babar	0.05%	0.04%	0.11%	0.00%
SINE	0.11%	0.06%	0.29%	0.80%
LTR	0.53%	0.62%	1.79%	2.50%
LTR/Copia	0.00%	0.00%	0.00%	0.00%
LTR/ERV1	0.00%	0.02%	0.01%	0.00%
LTR/Gypsy	0.22%	0.08%	1.28%	0.79%
Unknown	3.52%	3.42%	10.39%	12.33%
Satellite	0.16%	0.10%	0.00%	0.04%
Simple_repeat	19.50%	23.93%	6.90%	0.41%
Low_complexity	8.49%	9.49%	2.04%	0.33%

Supplementary Table 11. Simple sequence repeats in different species*.

	Species	Common name	Percent (%)
**Plants**	*B. distachyon*	Purple false brome	1.89
	S. bicolor	Sorghum	2.49
	O. sativa	Asian rice	2.9
	Z. mays	Maíz	0.86
	A. thaliana	Thale cress	0.45
	T. urartu	Einkorn wheat	1.21
**Vertebrates**	*F. peregrinus*	Peregrine	0.01
	D. rerio	Zebrafish	0.28
	L. crocea	Large yellow croaker	1.72
	M. musculus	Mouse	1.56
	T. rubripes	Tiger puffer	1.74
	H. sapiens	Human	0.84
**Insects**	*B. mori*	Silkworm	0.47
	L. migratoria	Locust	0.2
	D. melanogaster	Fruit fly	1.08
	A. gambiae	Mosquitoes	1.08
	P. humanus	Human lice	10.52
	A. mellifera	Cape honey bee	1.92
**Other invetebrates**	*C. elegans*	Roundworm	0.35
	H. robusta	Leech	6.36
	C. teleta	Polychaete	1.09
	C. gigas	Pacific oyster	0.72
	L. gigantea	Owl limpet	0.55
	L. vannamei	Pacific white shrimp	23.93
	D. pulex	Water flea	0.78
	I. scapularis	Blacklegged ticks	0.54
	T. castaneum	Beetle	0.24
	S. maritima	Centipede	0.80
	T. urticae	Mite	0.32
	N .vectensis	Sea Anemone	0.58
**Fungi**	*N. crassa*	Red bread mold	0.82
	S. cerevisiae	Yeast	0.36
	T. pseudonana	Marine centric diatom	0.23

* This table was update from our previous work (Zhang et al., 2019).

Supplementary Table 12. Comparison of SSRs among six crustacean genomes.*

SSR	Fchi	Lvan	Esin	Pvir	Phaw	Dpul
A	0.36%	0.35%	0.14%	0.00%	0.02%	0.06%
C	0.04%	0.04%	0.02%	0.19%	0.00%	0.02%
AT	5.51%	10.21%	1.01%	0.01%	0.04%	0.01%
GC	0.00%	0.00%	0.00%	0.00%	0.00%	0.00%
AC	4.18%	4.76%	1.14%	0.05%	0.01%	0.06%
AG	4.66%	4.53%	2.12%	0.02%	0.00%	0.07%
AAT	1.45%	0.88%	0.38%	0.01%	0.04%	0.02%
AAG	0.18%	0.15%	0.14%	0.00%	0.00%	0.08%
AAC	0.07%	0.06%	0.07%	0.01%	0.00%	0.04%
ATC	0.25%	0.17%	0.05%	0.00%	0.00%	0.01%
AGG	0.41%	0.28%	0.50%	0.01%	0.00%	0.01%
ACT	0.08%	0.07%	0.31%	0.00%	0.01%	0.01%
ATAG	0.36%	0.43%	0.07%	0.00%	0.00%	0.00%
ATAC	0.34%	0.31%	0.03%	0.00%	0.00%	0.01%
AGGG	0.19%	0.23%	0.03%	0.00%	0.00%	0.00%
ACAG	0.16%	0.14%	0.06%	0.00%	0.01%	0.00%
AAAG	0.08%	0.12%	0.03%	0.00%	0.00%	0.01%
AAAT	0.05%	0.09%	0.03%	0.01%	0.02%	0.01%
AACCT	0.01%	0.07%	0.01%	0.02%	0.02%	0.01%
AAACC	0.00%	0.00%	0.01%	0.00%	0.00%	0.00%
AAAAG	0.02%	0.04%	0.05%	0.00%	0.00%	0.01%
AGGGG	0.03%	0.03%	0.00%	0.00%	0.00%	0.00%
AATGAT	0.07%	0.06%	0.00%	0.00%	0.00%	0.00%
AGAGGG	0.06%	0.08%	0.00%	0.00%	0.00%	0.00%

* The SSR content lower than 0.005% were shown as 0.00% in the table. The crustaceans used for analysis were *Fenneropenaeus chinensis* (Fchi), *Litopenaeus vannamei* (Lvan), *Eriocheir sinensis* (Esin), *Procambarus virginalis* (Pvir), *Parhyale hawaiensis* (Phaw), *Daphnia pulex* (Dpul).

Supplementary Table 13. The genome resources of penaeid shrimp species*.

**Species**	**Read length**	**Total Bases**	**Accession ID**
Fenneropenaeus chinensis	100_100	138.08 Gb	This study
Fenneropenaeus merguiensis	100_100	671.9 Mb	DRR122407
Fenneropenaeus indicus	150_150	298.25 Gb	SRR7983095-SRR7983101
Litopenaeus vannamei	100_100	213.09 Gb	(Zhang et al., 2019)
Marsupenaeus japonicus	150_150	127.52 Gb	SRR5620465,SRR5620466
Penaeus monodon	150_150	127.30 Gb	SRR5620467,SRR5620468
Metapenaeus ensis	100_100	1.21 Gb	DRR122410
Metapenaeus joyneri	150_150	808.6 Mb	DRR122411

*The genome resources of these penaeid shrimps were downloaded from NCBI SRA database. These data were paired-end reads that sequenced by Illumina platform. Unlike the other species belong to genus of *Penaeus*, *Metapenaeus ensis* and *Metapenaeus joyneri* are two species belong to the genus of *Metapenaeus*, which set as the control for the comparative analyses.

Supplementary Table 14. The transcriptome data of the two shrimp species under low-salinity stress*.

Library	Clean Data(bp)	Q20(%)	Q30(%)
Lv30‰-1	6540180266	6356157872 (97.19%)	6060403044 (92.66%)
Lv30‰-2	7330527332	7135199179 (97.34%)	6823001829 (93.08%)
Lv30‰-3	9929173692	9646161607 (97.15%)	9128378844 (91.93%)
Lv9‰-1	6824579913	6616006093 (96.94%)	6242030064 (91.46%)
Lv9‰-2	7121769862	6912915227 (97.07%)	6580523625 (92.40%)
Lv9‰-3	8903596533	8596307550 (96.55%)	8070106756 (90.64%)
Lv3‰-1	7285650546	7069634057 (97.04%)	6724836569 (92.30%)
Lv3‰-2	6381343325	6188592202 (96.98%)	5840857071 (91.53%)
Lv3‰-3	6180832598	5992036453 (96.95%)	5652077168 (91.45%)
Fc30‰-1	5712801449	5550368175 (97.16%)	5251066243 (91.92%)
Fc30‰-2	6971553491	6770648240 (97.12%)	6405750011 (91.88%)
Fc30‰-3	7279236695	7087640931 (97.37%)	6742802818 (92.63%)
Fc9‰-1	9757990404	9504971840 (97.41%)	9040327882 (92.65%)
Fc9‰-2	8975262091	8740257669 (97.38%)	8311468741 (92.60%)
Fc9‰-3	8263133206	8082615754 (97.82%)	7732612635 (93.58%)
Fc3‰-1	9400226904	9153990360 (97.38%)	8707871370 (92.63%)
Fc3‰-2	8603564274	8396738698 (97.60%)	8010229679 (93.10%)
Fc3‰-3	10900184232	10653052050 (97.73%)	10188486425 (93.47%)

*Comparative transcriptomics analyses were performed on both shrimp species under the salinity of 30‰, 9‰ and 3‰. Three replicates were conducted per salinity.

Supplementary Table 15. The differentially expressed gene numbers in pairwise comparison of different levels of salinities.

Pair	DiffGene(Up)	DiffGene(Down)	All DiffGene
Lv_30‰-VS-Lv_9‰	1114	168	1282
Lv_30‰-VS-Lv_3‰	743	713	1456
Lv_9‰-VS-Lv_3‰	12	100	112
Fc_30‰-VS-Fc_9‰	13	10	23
Fc_30‰-VS-Fc_3‰	758	344	1102
Fc_9‰-VS-Fc_3‰	2735	789	3524

Supplementary Table 16. The differential expressed gene numbers in the pathways related to amino acid and lipid metabolisms.

Compare lib	*L. vannamei*	*F. chinensis*
**Glycine, serine and threonine metabolism**		
all*	51	63
30‰~3‰	11	9
30‰~9‰	8	0
9‰~3‰	4	8
**Cysteine and methionine metabolism**		
all	48	45
30‰~3‰	5	4
30‰~9‰	4	0
9‰~3‰	6	3
**Taurine and hypotaurine metabolism**		
all	8	9
30‰~3‰	4	2
30‰~9‰	1	0
9‰~3‰	0	3
**Glycerolipid metabolism**		
all	60	61
30‰~3‰	8	3
30‰~9‰	9	1
9‰~3‰	3	2
**Glycerophospholipid metabolism**		
all	86	79
30‰~3‰	8	8
30‰~9‰	10	0
9‰~3‰	1	5

* "all" indicates the total number of genes of the correspond pathways identified in the shrimp genomes.

Supplementary Table 17. The clean reads and mapping reads of ATAC-seq.

Sample	Clean reads	Mapping reads	Mapping rate (%)
LvHC1	261,584,236	215,436,909	82.36%
LvHC2	247,117,160	188,863,104	76.43%
LvHL1	345,537,552	300,198,312	86.88%
LvHL2	193,377,110	168,814,603	87.30%
FcHC2	135,203,142	114,237,232	84.49%
FcHC1	183,498,426	157,804,231	86.00%
FcHL1	129,496,364	113,998,385	88.03%
FcHL2	155,839,756	136,117,711	87.34%

Supplementary Table 18. The ATAC peaks identified in the two shrimp genomes.*

Sample	Peak number	Total length (bp)	Average length (bp)
LvHC1	300,000	195,357,243	651.19
LvHC2	300,000	191,071,458	636.9
LvHL1	300,000	149,052,689	496.84
LvHL2	300,000	178,886,046	596.29
FcHC2	300,000	203,608,308	678.69
FcHC1	300,000	172,695,793	575.65
FcHL1	300,000	174,325,187	581.08
FcHL2	300,000	186,439,215	621.46

* The top 300,000 peaks were selected for the following analyses.

Supplementary Table 19. The differential ATAC peaks under low-salinity stress.*

**Peaks**	***L. vannamei***	***F. chinensis***
30‰	98,967	106,314
3‰	112,604	149,442
1.00E-02	60,513	86,922
1.00E-03	17,258	21,587
1.00E-05	3,291	4,294
1.00E-10	575	1,588

* 30‰ and 3‰ indicates the total peaks identified in the control and low-salinity samples. 1.00E-02 to 1.00E-10 indicates the differential peak numbers with *p* value lower than 1.00E-02 to 1.00E-10.
