# Supplementary material for: Simple sequence repeats drive genome plasticity and promote adaptive evolution in penaeid shrimp
Source: Commun Biol. 2021 Feb 11;4:186. doi: 10.1038/s42003-021-01716-y (PMC7878876; doi:10.1038/s42003-021-01716-y)
Supplement: Supplementary file 3 — Description of Additional Supplementary Files [file 42003_2021_1716_MOESM3_ESM.pdf]

Description of Additional Supplementary Files

File Name: Supplementary Data 1

Description:

Table SS1. GO enrichment analysis of the expanded gene families of two penaeid shrimp species.

Table SS2. KEGG enrichment analysis of the expanded gene families of two penaeid shrimp species.

Table SS3. The length of various SSRs of different arthropods.

Table SS4. The density of various SSRs of different arthropods.

Table SS5. The relative quantity of metabolites identified in the salinity of 30‰ and 3‰ in the two penaeid shrimp species.

Table SS6. The differentially regulated compounds under low-salinity pressure in F. chinensis.

Table SS7. The differentially regulated compounds under low-salinity pressure in L. vannamei.
