# Supplementary material for: Simple sequence repeats drive genome plasticity and promote adaptive evolution in penaeid shrimp
Source: Commun Biol. 2021 Feb 11;4:186. doi: 10.1038/s42003-021-01716-y (PMC7878876; doi:10.1038/s42003-021-01716-y)
Supplement: Supplementary file 5 — Reporting Summary [file 42003_2021_1716_MOESM5_ESM.pdf]

nature research

Corresponding author(s): COMMSBIO-20-2738A

Last updated by author(s): Jan 7, 2021

Reporting Summary

Nature Research wishes to improve the reproducibility of the work that we publish. This form provides structure for consistency and transparency in reporting. For further information on Nature Research policies, see our Editorial Policies and the Editorial Policy Checklist.

Statistics

For all statistical analyses, confirm that the following items are present in the figure legend, table legend, main text, or Methods section.

n/a	Confirmed	
☐	☒	The exact sample size (*n*) for each experimental group/condition, given as a discrete number and unit of measurement
☐	☒	A statement on whether measurements were taken from distinct samples or whether the same sample was measured repeatedly
☐	☒	The statistical test(s) used AND whether they are one- or two-sided<br>*Only common tests should be described solely by name; describe more complex techniques in the Methods section.*
☐	☒	A description of all covariates tested
☐	☒	A description of any assumptions or corrections, such as tests of normality and adjustment for multiple comparisons
☐	☒	A full description of the statistical parameters including central tendency (e.g. means) or other basic estimates (e.g. regression coefficient) AND variation (e.g. standard deviation) or associated estimates of uncertainty (e.g. confidence intervals)
☐	☒	For null hypothesis testing, the test statistic (e.g. *F*, *t*, *r*) with confidence intervals, effect sizes, degrees of freedom and *P* value noted<br>*Give P values as exact values whenever suitable.*
☐	☒	For Bayesian analysis, information on the choice of priors and Markov chain Monte Carlo settings
☐	☒	For hierarchical and complex designs, identification of the appropriate level for tests and full reporting of outcomes
☐	☒	Estimates of effect sizes (e.g. Cohen's *d*, Pearson's *r*), indicating how they were calculated

Our web collection on statistics for biologists contains articles on many of the points above.

Software and code

Policy information about availability of computer code

Data collection	No softwares and codes are used for data collection. The data used for comparative genomics analysis were directly downloaded from the NCBI (www.ncbi.nlm.nih.gov) and Rfam database (http://xfam.org/).
Data analysis	The softwares weused for data analysis include: Trimmomatic v.0.35, BluePippin v6.31, Jellyfish-0.3, wtdbg-1.2.8, SMRT Analysis v2.3.0, BWA v0.7.12, SSPACE 3.0, Bowtie 2.3.4.3, CEGMA v3, RepeatModeler Open-1.0, RepeatMasker 4.0.5, Trinity v2.6.5, TopHat v1.2.1, Cufflinks v2.2.1, Exonerate v2.2.0, Augustus v2.5.5, Tophat v2.1.1, EvidenceModeler (EVM) r03062010, InterProScan 5, HMMER-3.0a1, OrthoMCL v1.4, RAxML Workbench 1.0, MUSCLE 3.8.31, PAML v4.48a, MEGA 7, Genome Analysis Toolkit (GATK) 3.6,

For manuscripts utilizing custom algorithms or software that are central to the research but not yet described in published literature, software must be made available to editors and reviewers. We strongly encourage code deposition in a community repository (e.g. GitHub). See the Nature Research guidelines for submitting code & software for further information.

Data

Policy information about availability of data

All manuscripts must include a data availability statement. This statement should provide the following information, where applicable:

- Accession codes, unique identifiers, or web links for publicly available datasets
- A list of figures that have associated raw data
- A description of any restrictions on data availability

Genome sequence data that support the findings of this study have been deposited in NCBI GenBank with the accession number of JABKCB000000000 and BioProject accession codes of PRJNA627295. The F. chinensis genome and the annotation information can also be downloaded from the Shrimp Genome Database (http://www.genedatabase.cn/fch_genome.html).

Field-specific reporting

Please select the one below that is the best fit for your research. If you are not sure, read the appropriate sections before making your selection.

- [x] Life sciences
- [ ] Behavioural & social sciences
- [ ] Ecological, evolutionary & environmental sciences

For a reference copy of the document with all sections, see nature.com/documents/nr-reporting-summary-flat.pdf

Life sciences study design

All studies must disclose on these points even when the disclosure is negative.

Sample size	We sequenced the genome of a single male adult of F. chinensis, and performed Hi-C sequencing of L. vannamei. Besides, three samples per salinity (30‰, 9‰, 3%) were collected for RNA-seq, metabolome and ATAC-seq sequencing.
Data exclusions	No samples were excluded.
Replication	All attempts at replication were successful.
Randomization	All the samples were collected randomly for RNA-seq, metabolome and ATAC-seq sequencing.
Blinding	Blinding was not relevant.

Reporting for specific materials, systems and methods

We require information from authors about some types of materials, experimental systems and methods used in many studies. Here, indicate whether each material, system or method listed is relevant to your study. If you are not sure if a list item applies to your research, read the appropriate section before selecting a response.

Materials & experimental systems

n/a	Involved in the study	
x		Antibodies
x		Eukaryotic cell lines
x		Palaeontology and archaeology
	x	Animals and other organisms
x		Human research participants
x		Clinical data
x		Dual use research of concern

Methods

n/a	Involved in the study	
x		ChIP-seq
	x	Flow cytometry
x		MRI-based neuroimaging

Animals and other organisms

Policy information about studies involving animals; ARRIVE guidelines recommended for reporting animal research

Laboratory animals	Fenneropenaeus chinensis, QD-2010, adult male; Litopenaeus vanamei, Keihai No. 1, adult male.
Wild animals	The wild individuals of Fenneropenaeus chinensis were captured by the local fisherman in Qingdao, China. The eight wild individuals of Litopeneaus vannamei were captured by the local fisherman in Baja California Sur, Mexico. The samples were collected and put into the freezer for storage and then send to the lab for DNA extraction. The body weight of these eight shrimp were approximately 6 gram in body weight, among them four were female and the other four were male.
Field-collected samples	Adult shrimp of F. chinensis (6.8 ± 0.4 cm) and L. vannamei (7.2 ± 0.5 cm) were collected at the shrimp culture laboratory of the Institute of Oceanology Chinese Academy of Sciences (IOCAS) in Qingdao, and acclimated to a salinity of 30‰. The salinity was gradually reduced to 3‰, and the animals were allowed to acclimate to salinities of 9‰ and 3‰ for 24 hours. Then, hepatopancrea samples were collected from animals acclimated to salinities of 30‰, 9‰ and 3‰.
Ethics oversight	All animals were handled and treated according to the alpaca guidelines approved by the Animal Ethics Committee [2020(37)] at Institute of Oceanology, Chinese Academy of Sciences (Qingdao, Shandong, China).

Note that full information on the approval of the study protocol must also be provided in the manuscript.

Flow Cytometry

Plots

Confirm that:

- [x] The axis labels state the marker and fluorochrome used (e.g. CD4-FITC).
- [x] The axis scales are clearly visible. Include numbers along axes only for bottom left plot of group (a 'group' is an analysis of identical markers).
- [x] All plots are contour plots with outliers or pseudocolor plots.
- [x] A numerical value for number of cells or percentage (with statistics) is provided.

Methodology

Sample preparation	Appendages were collected from shrimp individuals, and mouse"KM" (genome size of 2.50 Gb) blood cells were used as internal standard. Briefly, samples of tissues were chopped with a razor blade in the buffer of PBS. 1 mL of the homogenized cell suspension was filtered through a 30 μm nylon filter, added with 12 μL of propidium iodide (50mg/mL), and stained with 2 μL of RNase (10 mg/mL) for 20 mins.
Instrument	BD FACSAria II
Software	BD FACSDiva3.0
Cell population abundance	1×10 6
Gating strategy	SSC×FSC to include shrimp hemocyte, PE-Texas Red-H×PE-Texas Red-W to include G0 stage cell

- [x] Tick this box to confirm that a figure exemplifying the gating strategy is provided in the Supplementary Information.
